# Prediction of Potential Distribution of Seven Plant Species of *Aster* (Asteraceae) Based on MaxEnt Model

**DOI:** 10.1002/ece3.71931

**Published:** 2025-09-30

**Authors:** Jinping Qin, Yanlong Wang, Xiaoli Wang, Yuan Ma, Ying Liu, Yushou Ma

**Affiliations:** ^1^ Qinghai Academy of Animal and Veterinary Science Qinghai University Xining Qinghai Province China; ^2^ Northwest Institute of Plateau Biology Chinese Academy of Sciences Xining Qinghai China; ^3^ State Key Laboratory of Plateau Ecology and Agriculture Qinghai University Xining Qinghai Province China

**Keywords:** *Aster*, climate change, MaxEnt model, species distribution, suitable habitat

## Abstract

*Aster* (Asteraceae) species as one of the traditional Tibetan medicinal plants in China have high useful medicinal and unique ornamental value; the market demand has been gradually increasing. In this study, seven species of *Aster* were selected from the Qinghai‐Tibet Plateau, and the MaxEnt model was used to investigate their potential distribution in China and the changes in their suitable habitat under future climate conditions based on the current survey and distribution data of specimens on the site and six to eight environmental variables. The results showed that temperature and precipitation were important limiting factors affecting the distribution of *Aster*, and Bio2, Bio3, and Bio10 were common environmental factors influencing the factors of *Aster* species. Under the current climate, the mainly potential distributed region of the seven *Aster* species in the Qinghai‐Tibet Plateau exists. Under projected future climate scenarios, the suitable habitats of *A. asteroides* and *A. diplostephioides* will shrink significantly, while those of *A. farreri* , *A. poliothamnus*, *A. souliei* , *A. tongolensis* , and *A. yunnanensis* var. *labrangensis* will expand accordingly. Environmental factors provide a large gain in predicting the distribution of *Aster* species. Among the environmental variables, isothermality (Bio3) induced the largest impact on SDM and contained the most useful information for *A. diplostephioides* (55.9%), *A. souliei* (41.5%) and *A. yunnanensis* var. *labrangensis* (27.1%), while *A. tongolensis* (27.9%) and *A. poliothamnus* (26.8%) were more significantly affected by the temperature seasonality (Bio4); *A. asteroides* (66.3%) and *A. farreri* (21%) were more significantly affected by the mean temperature of the warmest quarter (Bio10). The study findings suggest that the distribution range of seven species of *Aster* will be greatly impacted by climate change. This research helps identify the limiting factors affecting the natural distribution and potential suitable areas for *Aster* species, which can inform conservation efforts, plant introduction, acclimatization, domestication, and cultivation of *Aster*.

## Graphical abstract

In this study, seven species of Aster were selected from Tibet, and the Maxent model was used to investigate their potential distribution in China and the changes in their suitable habitat under future climate conditions based on the actual survey and distribution data of specimens on the site and 6–8 environmental variables; it indicated that Aster plant distribution is influenced by multiple environmental factors, particularly temperature and precipitation.

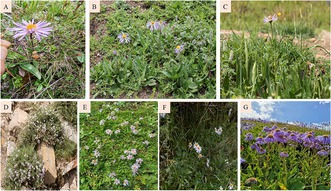

## 1 Introduction

**FIGURE 1:**
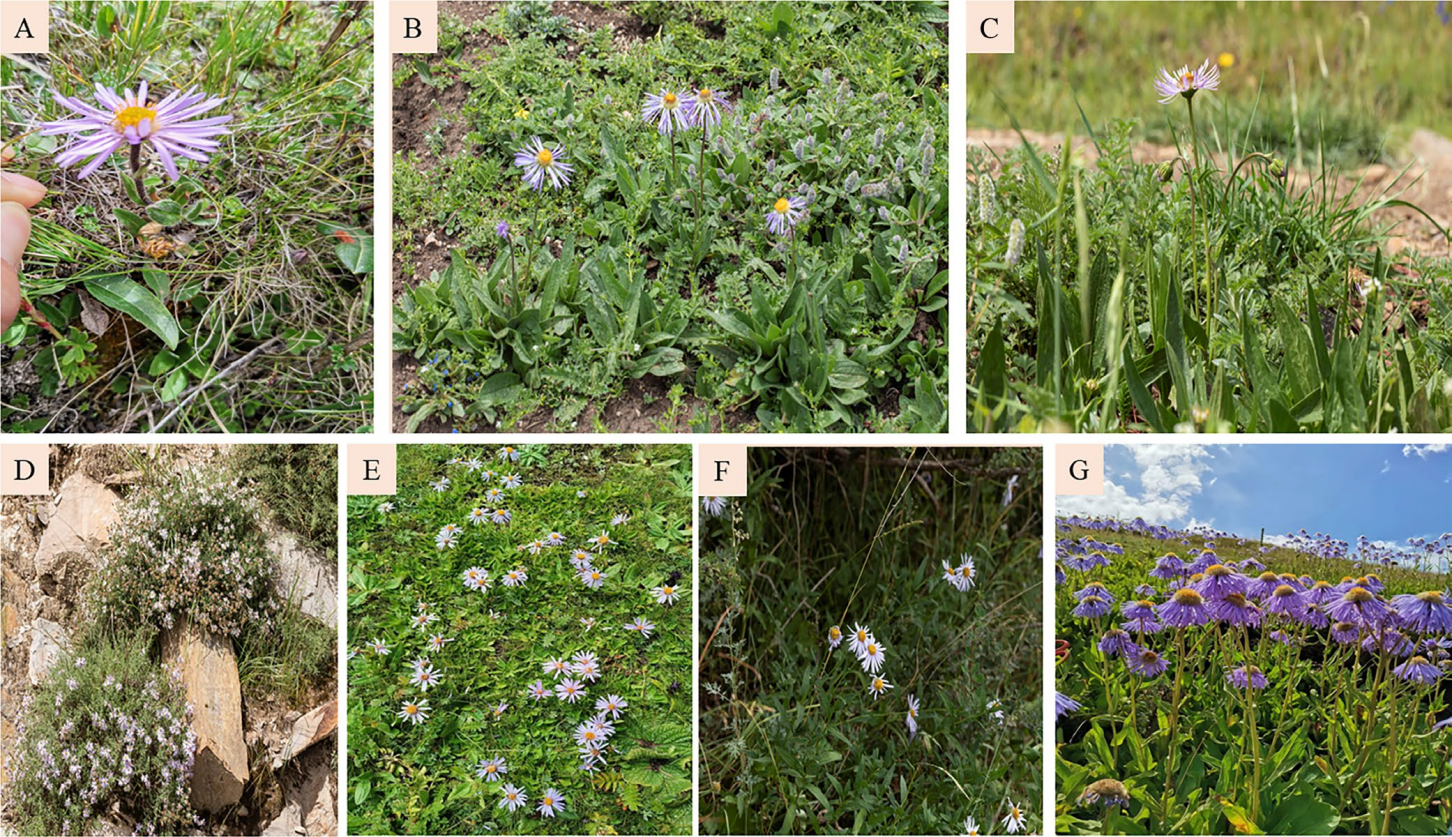
Seven species of *Aster* genus. (A) *Aster asteroides* , (B) *Aster diplostephioides* , (C) *Aster farreri* , (D) *Aster poliothamnus*, (E) *Aster souliei*, (F) *Aster tongolensis* , and (G) *Aster yunnanensis* var. *labrangensis*.

*Aster*, a genus in the Asteraceae family, *Aster* species include perennial herbs, subshrubs, and shrubs. Their stems are typically erect and branched, with alternate leaves that may be dentate or entire. The capitulum features narrow and elongated ligules of the ray florets, which are predominantly purple, blue, white, or light red, while the disc florets are yellow or purplish‐brown at the apex. Plants in the *Aster* genus possess well‐developed root systems (Lin and Chen 1985; Liu 1996) (Figure 1). *Aster species* have a rich history of medicinal use in China (Ying et al. 2016; Li et al. 2022). It is one of the traditional Chinese medicinal plants and the main source of Tibetan medicinal materials on the Qinghai‐Tibet Plateau (The Qinghai‐Tibet Plateau (QTP), situated in southwestern China, is the highest and largest plateau globally, averaging over 4000 m above sea level, and is often referred to as the ‘Roof of the World’. As the Earth's Third Pole, the QTP is characterized by its complex and diverse terrain, which plays a crucial role in global climate regulation and biodiversity conservation. Its vertically distributed vegetation zones form essential regional habitats that support remarkable endemism and represent a significant global biodiversity hotspot.) (An et al. 2009; Ying et al. 2016; Guo et al. 2022). There are approximately 250 species within the *Aster* genus; *Aster* species are recognized for their abundance in phytochemical and pharmacological components, including quercetin, flavonoids, saponins, chlorogenic acid, caffeic acid, and its derivatives (Lee et al. 2013; Park et al. 2015; Choi et al. 2017; He et al. 2019; Li et al. 2022); these compounds contribute to various therapeutic effects such as hypolipidemic activity (Kim et al. 2016), lung moisturization and expectorant properties (Lee et al. 2019), antibacterial and antiviral activities, diuretic effects (Schafhauser et al. 2019; Su et al. 2019), anti‐cancer potential, and anti‐inflammatory properties (Choi et al. 2014; Lim et al. 2021). The flowers of the *Aster* species exhibit predominantly blue‐purple hues with unique colors that make them suitable for landscape development in high‐altitude regions (Zuo 2016). In recent years, the market demand for *Aster* plants has witnessed a surge owing to their significant medicinal and ornamental value (Gempo et al. 2024), thereby necessitating research on the distribution of *Aster* and its potential changes under future climate change. The Qinghai‐Tibet Plateau is renowned for harboring numerous medicinal plants (Zhang and Chen 2003; Huang 2011; Wu et al. 2022). However, there is currently limited exploration of the natural distribution of *Aster* species on the Qinghai‐Tibet Plateau, with most studies focusing primarily on their medicinal value and chemical composition (An et al. 2009; Li et al. 2022). Furthermore, conducting field investigations to determine suitable areas for plant distribution is increasingly challenging due to the complex environmental conditions prevailing in this region (Yang et al. 2024). Despite the heavy reliance on wild germplasm resources for developing and utilizing the medicinal properties of *Aster* (Lee et al. 2019; He et al. 2019), large‐scale cultivation of Tibetan medicinal *Aster* remains inadequate in China and other regions, mainly due to slow progress in selecting appropriate varieties. As the annual demand for wild *Aster* resources continues to rise, it becomes imperative to consider complex environmental factors and climate change that influence plant distribution patterns.

Complex environmental factors and climate change influence the distribution of plants (Hu et al. 2020; Moraitis et al. 2019; Urban et al. 2013). Species distribution models (SDMs) effectively predict potentially suitable ranges based on comprehensive environmental conditions while estimating the relationship between species distribution and environmental factors through calculations (Booth et al. 2014; Atwater and Barney 2021; Guisan et al. 2014). Among these, the MaxEnt (Maximum Entropy) is frequently employed as a predictive tool for plant distribution modeling purposes (Li et al. 2019; Li et al. 2020; Cursach et al. 2020; Khan et al. 2022; Damaneh et al. 2022). For instance, Hou et al. utilized the maximum entropy model to predict the suitable environment and cultivation area of Pepino and assess its cultivation area and adaptability (Hou et al. 2023). Yang et al. projected the potential distribution of 12 endangered medicinal plants on the Qinghai‐Tibet Plateau (Yang et al. 2024). The MaxEnt model and ArcGIS were adopted to predict the potential geographical distribution of *Symphyotrichum subulatum* ( *S. subulatum* ) in China and the alterations of its suitable areas under future climate scenarios. Additionally, the relationship between the distribution of *S. subulatum* and environmental variables was also examined. The results indicated that the two environmental variables, namely the driest part of the month (Bio14) and the wettest part of the month (Bio13), were most closely associated with the suitable areas of *S. subulatum* (Li et al. 2023).

Currently, the research on the distribution of the Tibetan medicinal genus *Aster* in the Qinghai‐Tibet Plateau is relatively limited. Moreover, there is a dearth of studies concentrating on its potential distribution in suitable areas, the influence of environmental factors on its distribution, and how its distribution might change under future climate conditions. To obtain information regarding the distribution range of Tibetan medicinal *Aster* species and their distribution alterations under future climate change, this study selected seven *Aster* species distributed on the Qinghai‐Tibet Plateau and combined MaxEnt and SDM models to explore their suitable distribution ranges in China and their relationship with environmental factors and investigate the changes in the suitable distribution area under future climate change. The study provides the foundation for the protection, management, development, and utilization of *Aster* on the Qinghai‐Tibet Plateau.

## 2 Materials and Methods

### 2.1 Distribution Information Data of *Aster* Species

These seven *Aster* species were selected primarily due to their status as common endemic taxa in the Qinghai‐Tibet Plateau and their widespread use in Tibetan traditional medicine. As key components of local pharmacopeia, they serve critical roles in healthcare practices for plateau communities, underscoring the need to safeguard their ecological sustainability. The modeling extent was constrained to China's political boundaries to align with regional conservation priorities and data availability. While some species (e.g., *Aster farreri* ) may occur in neighboring regions like Nepal or Myanmar, this study focuses on China due to (1) the primary medicinal use and conservation needs within the country; (2) limitations in accessing high‐resolution occurrence data for extra‐national areas. The occurrence data for the seven species of *Aster* (Figure 1) in this study was collected through field surveys conducted in the source area of the Yellow River from 2020 to 2023. Additional information was sourced from a combination of literature, Flora of China, Flora of Qinghai, Flora of Tibet (Wu 1985), Digital Herbarium of China (https://www.cvh.ac.cn/) (Lin and Chen 1985; Liu 1996) and Global Biodiversity Information Institution (https://www.gbif.org/zh/) (Shi, Zhu, and Wang 2023).

After the removal of duplicate records and records without precise geographic coordinates of specimens, a total of 806 (Table S1) records on the natural distribution of *Aster* species in China were collected (61 *Aster asteroids* (*A*. *asteroids*), 117 *Aster diplostephioides* ( *A. diplostephioides* ), 131 *Aster farreri* ( *A. farreri* ), 75 *Aster poliothamnus* (*A*. *poliothamnus*), 210 *Aster souliei* ( *A. souliei* ), 128 *Aster tongolensis* ( *A. tongolensis* ), and 84 *Aster yunnanensis* var. *labrangensis* ( *A. yunnanensis* var. *labrangensis*)). In this study, we selected 669 records (60 *A. asteroides* , 92 *A. diplostephioides* , 111 *A. farreri* , 60 *A. poliothamnus*, 169 *A. souliei* , 110 *A. tongolensis* , and 67 *A. yunnanensis* var. *labrangensis*)based on the recorded information of the samples and avoiding overfitting of the model, taking the latitude and longitude range of 2.5′*2.5′ as the effective distribution point of the population.

### 2.2 Climate Parameters

Current climate variables and future climate data (2040–2060 and 2080–2100) were downloaded from the World Climate Database Website (http://www.worldclim.org/) (Fick and Hijmans 2017; Wang et al. 2023). Using the BCC‐CSM2‐MR model, which has strong simulation capabilities in China (Shi, Wang, et al. 2023; Guan et al. 2022), four CO_2_ emission scenarios, ssp126, ssp245, ssp370, and ssp585, were selected. Nineteen bioclimatic variables were included (see Table S2: Bio01 ~ Bio19); the spatial resolution was 2.5′, and the geographic coordinate system of the map was the projection coordinate system GCS_WGS_1984.

When building the species distribution model, the multicollinearity of the environmental variables leads to an overfitting of the species distribution model, which reduces the accuracy of the prediction results (Amiri et al. 2020). We used Pearson correlation to analyze the correlation between *Aster* species distribution data and climate variables (Supporting Information 2: Figure 23–29). To address collinearity, variables with |*r*| ≥ 0.8 were filtered using Pearson correlation analysis. For collinear pairs, the variable with higher ecological relevance and Jackknife‐derived importance (Wei et al. 2018) was retained. Remaining variables (|*r*| < 0.8) were further evaluated using MaxEnt's Jackknife tests, with those exhibiting higher contribution rates and clear ecological links (e.g., temperature/precipitation parameters) selected for modeling. This process yielded six to eight variables per Aster species, aligning with protocols for balancing statistical rigor and biological plausibility in niche modeling.

### 2.3 Geographical Distribution Mapping

**FIGURE 2:**
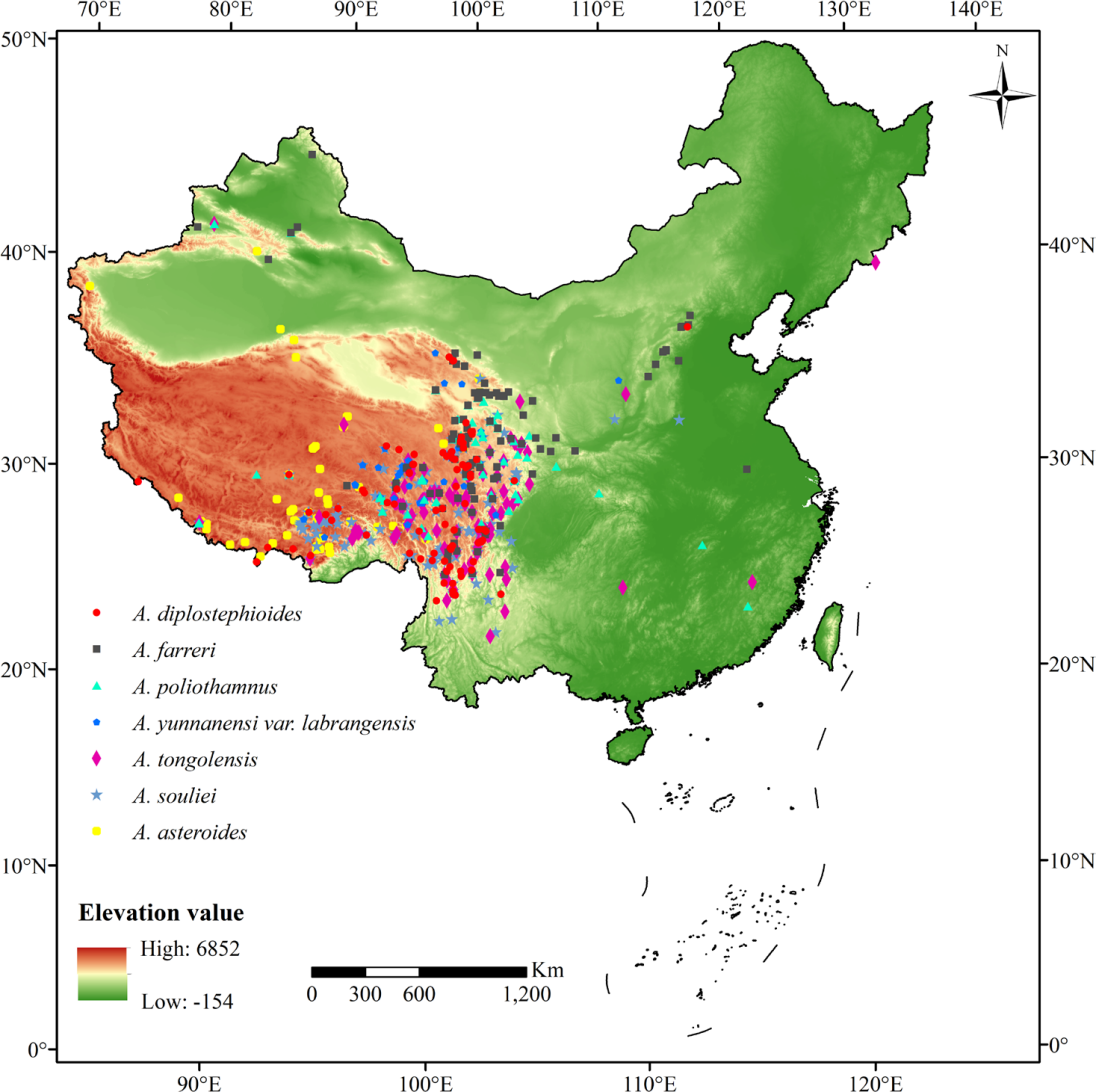
Geographic distribution records of the seven *Aster* species used in the study (pre‐modeling data). *Aster asteroids* (60), *Aster diplostephioides* (92), *Aster farreri* (111), *Aster poliothamnus* (60), *Aster souliei* (169), *Aster tongolensis* (110), *Aster yunnanensis* var. *labangensis* (67).

The effective distribution data of the finally selected *Aster* species were imported into ArcGIS software, and the actual distribution map was drawn based on the vector map of administrative districts of Chinese provinces (at a scale of 1∶4,000,000) (Figure 2). The ArcGIS v10.4 software was used to clip the climate variables, and the clipping area was the China region.

### 2.4 Model Parameter Optimization and Model Construction

To improve the accuracy and reliability of the prediction, the kuenm script of R 3.6.3 (Cobos et al. 2019; R Core Team 2020) was used in this study to optimize the regularization multiplier (RM) and feature class (FC) parameters of the MaxEnt model. The model parameters were determined based on the species and their data results (Kong et al. 2019). RM and FC parameters have an important influence on the MaxEnt model, where the value of the RM parameter is usually 0–4 (Gao et al. 2023) and FC parameters (Phillips et al. 2006) are mainly composed of linear features (L), quadratic features (Q), hinge features (H), product features (P) and threshold features (T). In this study, RM was set to 0–4 in MaxEnt and increased by 0.5 each time (Yan et al. 2021). Thirty‐one feature combinations were assumed, totaling 279 parameter combinations. After testing delta AICc (When delt. AICc is minimum) and training omission rate (the training omission rate was smaller) (Yang et al. 2022; Shi et al. 2021; Zhao et al. 2021). Finally, the models with the lowest delta AICc values were selected among the significant and low‐omission candidate models. The parameters of the optimal model were: *A. yunnanensis* var. *labrangensis* (RM:2.5, FC: LQPTH), *A. diplostephioides* (RM:2.5, FC: QTH), *A. farreri* (RM:0.5, FC: P), *A. souliei* (RM:1.5, FC: QPT), *A. asteroides* (RM:3.5, FC: LQPTH), *A. poliothamnus* (RM:1.5, FC: LQT) and *A. tongolensis* (RM:3.5, FC: LQPTH).

The *Aster* species distribution point data and the clipped environmental factors layer were imported into the MaxEnt software; the optimized model parameters were selected, 75% of the points were randomly selected by the software for modeling, and 25% of the points were verified for the model (Hu et al. 2014). The maximum number of iterations was set to 1000, and Bootstrap repeated the calculation 10 times. The response curves and Jackknife were used to analyze the relationship between the environmental factors and the distribution of *Aster*. The Jackknife tool was used to determine the effects of individual environmental variables on the distribution of each *Aster species* (Li et al. 2019). The response curves were used to evaluate the optimal climatic conditions for seven *Aster* species. The Receiver Operating Characteristic Curve (ROC curve) analysis method was applied to verify the accuracy of the distribution results of suitable areas of *Aster* species predicted by the MaxEnt software; the area under the ROC curve is the AUC value. As an accuracy indicator for the predictive ability of the model (Ma et al. 2022; Xu et al. 2020), the AUC value ranges from 0.5 to 1. The closer the AUC value is to 1, the better the prediction result (Yang et al. 2024). The AUC values predicted by the model in this study for seven *Aster* species were all greater than 0.9 (Figure S1).

### 2.5 Classification of Suitable Habitats

The constructed model was imported into ArcGIS software, and according to the natural breaks (Jenks) method of the reclassification tool (Poirazidis et al. 2019; Shi, Zhu, and Wang 2023), the potential distribution areas of seven *Aster* species in China were categorized into the following four classes: unsuitable area, low suitable area, medium suitable area, and highly suitable area (Yan et al. 2020; Shi, Wang, et al. 2023).

Continuous MaxEnt outputs (0–1 suitability scores) were converted to binary maps using the 10th percentile training presence threshold. This method retains 90% of species occurrences within the suitable area, balancing the minimization of known population exclusion and overprediction. Threshold values were species‐specific (Table S4): *A. yunnanensis* var. *labrangensis* (0.225), *A. diplostephioides* (0.2585), *A. farreri* (0.2117), *A. souliei* (0.1547), *A. asteroides* (0.3969), *A. poliothamnus* (0.2605), and *A. tongolensis* (0.223). Sensitivity analyses comparing multiple thresholds (minimum training presence, maximum sensitivity plus specificity) are provided in Table S4.

### 2.6 Changes of Suitable Habitat Area and Centroid

To further investigate the trends in the distribution of seven Tibetan *Aster* species, the centroid of current and future climatic distribution regions was calculated using the SDMtoolbox v2.4 based toolkit (Brown et al. 2017). First, the ASC file generated by the MaxEnt model under different emission scenarios was imported into ArcGIS v.10.4, and the spatial distribution pattern and centroid change of the suitable habitat area of each *Aster* species were analyzed and mapped using SDMtoolbox. The area of suitable habitat for each *Aster* species under different climate scenarios was calculated, and the shrinkage and expansion of the suitable distribution area of *Aster* under future climate conditions was analyzed. To further analyze the changes in the distribution centroid of *Aster* under the current and future climate change scenarios, ArcGIS was used to narrow down the distribution area of suitable habitat to a single central point in different time periods, and the changes in the location of the distribution centroid were used to reflect the changes in the direction of the total suitable area and the highly suitable area of *Aster*, and to evaluate the migration distance of the suitable habitat area.

## 3 Results

### 3.1 Distribution Status of Seven *Aster* Species

Based on the inventory of *Aster* species between 2020 and 2023, the effective distribution points of seven species of *Aster* were worked out by combining literature data and sample database query methods. The results showed that seven species of *Aster* are mainly distributed in the eastern and southeastern parts of the Qinghai‐Tibet Plateau in China (Figure 2). *A. yunnanensis* var. *labrangensis* is mainly distributed in Yushu City and Nangqian County of Yushu Tibetan Autonomous Prefecture, Maqin County and Dari County of Guoluo Tibetan Autonomous Prefecture, Qinghai Province. *A. diplostephioides* is most common in Maqin, Dari, and Banma counties in Guoluo Prefecture, Qinghai Province, and Batang, Litang, and Baiyu counties in Garze Tibetan Autonomous Prefecture, Sichuan Province. *A. farreri* is mainly distributed in the eastern part of the Qinghai‐Tibet Plateau, especially in the southeast of Qinghai Province and the northwest of Sichuan Province. *A. souliei* is mainly distributed in the southern part of the Qinghai‐Tibet Plateau, in Yushu and Guoluo Prefectures of Qinghai Province, in Garze Tibetan Autonomous Prefecture of Sichuan Province, and in Nyingchi and Qamdo City of Tibet Autonomous Region. The distribution of *A. asteroides* is sparse and scattered, although it is relatively dense in Qamdo City of Tibet Autonomous Region and Guoluo Prefecture in Qinghai Province. The distribution areas of *A. poliothamnus* are mainly concentrated in the prefecture of Hainan in Qinghai Province and the Qamdo City of the Tibet Autonomous Region. The distribution areas of *A. tongolensis* are mainly concentrated in the Garze Tibetan Autonomous Prefecture and the Aba Tibetan Autonomous Prefecture of Sichuan Province.

### 3.2 ROC Curve Detection of MaxEnt Model Prediction Accuracy

In this study, the ROC curve was determined by running the average value of the model 10 times. According to the prediction results simulated by the MaxEnt model, the AUC values of *A. asteroids*, *A. diplostephioides* , *A. farreri* , *A. poliothamnus*, *A. souliei* , *A. tongolensis* , and *A. yunnanensis* var. *labangensis* were 0.903, 0.959, 0.923, 0.911, 0.951, 0.944, and 0.973, respectively. The AUC values of the ROC curve training set of seven *Aster* species were all greater than 0.90. The MaxEnt model was used to predict the potential geographic distribution of seven *Aster* species in China with high accuracy and reliability (Figure S1).

### 3.3 Environmental Influence Factors

**FIGURE 3:**
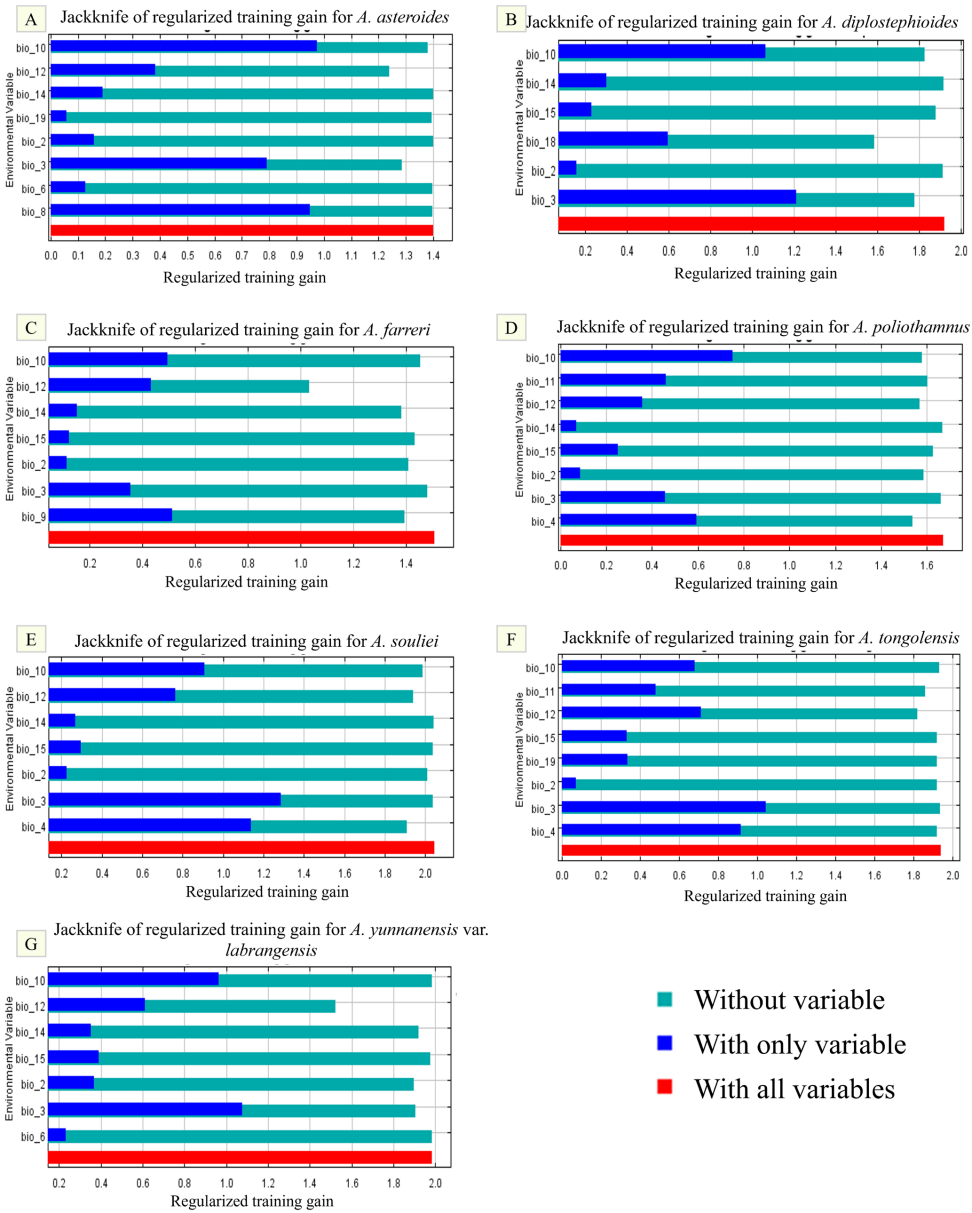
Training gain of environmental factors in forecasting the distribution of *Aster* species using MaxEnt. (A) *A. asteroides* , (B) *A. diplostephioides* , (C) *A. farreri* , (D) *A. poliothamnus*, (E) *A. souliei* , (F) *A. tongolensis* , and (G) *A. yunnanensis* var. *labrangensis*.

The Jackknife test (Figure 3) of AUC values revealed that Bio3, Bio10, and Bio12 have a greater influence on the distribution prediction model of *A. yunnanensis* var. *labrangensis* distribution, with a cumulative contribution rate of 81.2%. Bio9, Bio10, and Bio12 are the major influencing factors in its distribution prediction model, accounting for a cumulative contribution rate of 66.2%. In the case of *A. diplostephioides, Bio3, Bio10*, and Bio18 play a significant role in its distribution prediction model, with a cumulative contribution rate as high as 98.2%. For *A. souliei* , the key factors affecting its distribution prediction model are Bio3, Bio4, and Bio10, contributing cumulatively 78.4%. Bio10, Bio8, and Bio3 are the primary influencers of the distribution prediction model for *A. asteroides* , with a cumulative contribution rate of 85.1%. For *A. poliothamnus*, Bio10, Bio4, and Bio11 have a substantial impact on its distribution prediction model, showing a cumulative contribution rate of 73.5%. Additionally, Bio3, Bio4, and Bio12 are the main factors influencing the distribution prediction model of *A. tongolensis* , with a cumulative contribution rate of 67.1% (Table S3).

Among these, the three environmental factors (Bio3, Bio10 and Bio9) are a great gain for *Aster* species distribution prediction models. Bio3 (Isothermality), when used independently, has the greatest impact on the models predicting the distribution of *A. yunnanensis* var. *labrangensis, A. diplostephioides, A. souliei*, and *A. tongolensis*, containing the most valuable information. Bio10 (Mean Temperature of Warmest Quarter) has the most influence on the prediction models for the distribution of *A. asteroides* and *A. poliothamnus*, indicating that it holds more useful information for predicting the potential distributions of these two species. Bio9 (Mean Temperature of Driest Quarter) is the most influential factor in predicting the distribution of *A. farreri* , suggesting that it contains unique information not provided by other environmental factors for this species. Overall, these Bio3, Bio10, and Bio9 are key factors influencing the distribution and adaptability of the *Aster*.

### 3.4 Potential Distribution of Seven *Aster* Species in China

Based on the prediction results of the optimized MaxEnt model, GIS was used to generate distribution maps for seven *Aster* species under current climatic conditions. The maps identify potential suitable habitats for each species across different suitability grades, as illustrated in Figures S2–S8. The red area is a high suitability area, the orange area is a medium suitability area, the blue area is a low suitability area, and the white area is not a suitable area. According to Figures S2–S8, the seven species of *Aster* in this study are mainly distributed in the Qinghai–Tibet Plateau in China.

*Aster asteroides* is predominantly found in northwest China, with distribution across much of the Qinghai‐Tibet Plateau except for the Qaidam Basin. This plateau serves as the primary habitat for *A. asteroides* , covering 27.23% of China's total territory (Figure S2 and Table 1). Based on current climate conditions, the high suitability area for *A. asteroides* is estimated to be 74.85 × 10^4^ km^2^, concentrated in regions such as Yushu and Guoluo Tibetan Autonomous Prefectures in Qinghai Province, Qamdo and Nyingchi Cities in the Tibet Autonomous Region, and Garze and Aba Tibetan Autonomous Prefectures in Sichuan Province. The medium suitability area for *A. asteroides* spans Qinghai Province, the Tibet Autonomous Region, Sichuan Province, Gansu Province, and Xinjiang Uygur Autonomous Region, covering an estimated 75.55 × 10^4^ km^2^. Conversely, the low suitability area for *A. asteroides* encompasses much of the Tibet Autonomous Region, Qinghai Province, Yunnan Province, Gansu Province, with some areas in Shanxi Province and Xinjiang Uygur Autonomous Region. According to MaxEnt predictions, the low suitability area for *A. asteroides* is extensive, totaling 110.96 × 10^4^ km^2^.

**TABLE 1:** The area of each suitable area of seven *Aster* species under the current climate situation.

Species	Low suitable area (×10^4^ km^2^)	Medium suitable area (×10^4^ km^2^)	High suitable area (×10^4^ km^2^)	Total suitable area (×10^4^ km^2^)
*Aster asteroides*	110.96	75.55	74.85	261.36
*Aster diplostephioides*	68.53	41.2	45.65	155.38
*Aster farreri*	221.53	83.5	36.96	341.99
*Aster poliothamnus*	150.48	61.91	35.32	247.71
*Aster souliei*	65.86	38.23	35.57	139.66
*Aster tongolensis*	103.96	43.67	30.13	177.75
*Aster yunnanensis* var. *labrangensis*	95.05	38.32	32.59	165.96

Under the current climate conditions, approximately 16.19% of China's land area is deemed suitable for *Aster diplostephioides* (Figure S3 and Table 1), with the highest suitability found in the southern part of the Qinghai‐Tibet Plateau. This region includes Garze Tibetan Autonomous Prefecture, Aba Tibetan Autonomous Prefecture in Sichuan Province, and Qamdo Prefecture in Tibet Autonomous Region, with smaller areas of high suitability in Qinghai and Yunnan Provinces totaling about 45.65 × 10^4^ km^2^. Medium suitability areas for *A. diplostephioides* are predominantly in Tibet Autonomous Region and Qinghai Province, with some in Yunnan and Sichuan Provinces, covering an average area of about 41.20 × 10^4^ km^2^. Conversely, most parts of Yunnan Province, southern Gansu Province, central Qinghai Province, and southern Tibet Autonomous Region are considered low suitability areas for *A. diplostephioides* . Additionally, there are also a few low‐suitability areas for *A. diplostephioides* in central Taiwan Province, totaling an area of 68.53 × 10^4^ km^2^.

*Aster farreri* exhibits a broad range of suitable habitats in China, particularly in northern regions. Currently, its suitable habitat area covers 35.62% of China's land area expanse (Figure S4 and Table 1). The high suitable habitat area was predicted to be 36.96 × 10^4^ km^2^, predominantly situated in the southeastern portion of the Qinghai‐Tibet Plateau, encompassing areas in Qinghai Province, southern Gansu Province, northwestern Sichuan Province, and eastern Tibet Autonomous Region. The medium suitable habitat area for *A. farreri* is primarily found in Qinghai Province, Gansu Province, Ningxia Hui Autonomous Region, Shaanxi Province, Shanxi Province, Sichuan Province, and Tibet Autonomous Region, covering an area of 83.50 × 10^4^ km^2^. Conversely, the low suitability area for *A. farreri* is mainly concentrated in the northern and northwestern regions of China, spanning a total area of 221.53 × 10^4^ km^2^. Overall, the species demonstrates a wide range of suitable habitats and a considerable latitude span.

Under the current climate conditions, the suitable habitat for *Aster poliothamnus* in China spans latitudes from 20° to 43° N, covering approximately 25.80% of China's territorial area (Figure S5 and Table 1). The highly suitable areas are primarily found in the Qinghai‐Tibet Plateau, encompassing provinces such as Sichuan, Qinghai, Gansu, and Tibet, with some additional areas in Yunnan and Ningxia. These highly suitable areas total 35.32 × 10^4^ km^2^, representing 3.68% of China's land area. The medium suitable areas for *A. poliothamnus* are situated in provinces like Sichuan, Qinghai, Gansu, Tibet, Yunnan, Shaanxi, Ningxia, Henan, Hubei, Guizhou, and Shanxi, although these areas are less extensive, covering 61.91 × 10^4^ km^2^. Conversely, the low suitable areas for *A. poliothamnus* are widespread across the country, with a total area of 150.48 × 10^4^ km^2^.

Under the current climate conditions, the suitable habitat for *Aster souliei* is relatively small, primarily concentrated in the southwest of China, covering 14.55% of China's territorial area (Figure S6 and Table 1). The high habitat area was predicted to be 35.57 × 10^4^ km^2^, mainly situated in Aba Prefecture and Ganzi Prefecture in Sichuan Province, Qamdo Prefecture in Tibet, and Gannan Tibetan Autonomous Prefecture in Gansu Province. The medium suitable areas of *A. souliei* are predominantly found in Qinghai Province, Sichuan Province, and Tibet Autonomous Region, totaling 38.23 × 10^4^ km^2^. Areas with low suitable areas for *A. souliei* are primarily located in Qinghai Province, Sichuan Province, Gansu Province, Yunnan Province, and Tibet Autonomous Region. Additionally, there are scattered low suitability areas in Ningxia Hui Autonomous Region, Shaanxi Province, and Guizhou Province, covering a total area of 65.86 × 10^4^ km^2^.

*Aster tongolensis* is widely distributed in various regions of our country, with the majority found in the southwest and central areas, covering 18.52% of China's territorial area (Figure S7 and Table 1). The high suitability regions are concentrated in Sichuan Province and Tibet Autonomous Region, totaling 30.13 × 10^4^ km^2^. Medium suitability regions are primarily located in Yunnan Province, Sichuan Province, Tibet Autonomous Region, Gansu Province, and Qinghai Province, covering 43.67 × 10^4^ km^2^. Conversely, the low suitability areas of *A. tongolensis* can be found in Yunnan Province, Qinghai Province, Tibet Autonomous Region, Sichuan Province, Gansu Province, Ningxia Hui Autonomous Region, Shanxi Province, Shaanxi Province, Henan Province, and Hubei Province, with a total area of 103.96 × 10^4^ km^2^.

The MaxEnt model was utilized to predict the potential geographic range of *Aster yunnanensis* var. *labrangensis* under current climate conditions. The distribution area of *A. yunnanensis* under these conditions is primarily situated in the southeast of the Qinghai–Tibet Plateau, covering a total suitable area of 165.96 × 10^4^ km^2^, which represents 17.29% of the study area (Figure S8 and Table 1). The latitude range for *A. yunnanensis* var. *labrangensis* spans from 25° to 35°. Regions with high suitability are predominantly found in Yushu Tibetan Autonomous Prefecture and Guoluo Tibetan Autonomous Prefecture in Qinghai Province, Qamdo City in Tibet Autonomous Region, and Garze Tibetan Autonomous Prefecture and Aba Tibetan Autonomous Prefecture in Sichuan Province, totaling approximately 32.59 × 10^4^ km^2^. The medium suitable area for *A. yunnanensis* var. *labrangensis* is mainly concentrated in Qinghai Province, Sichuan Province, and Tibet Autonomous Region, with smaller portions in Yunnan Province and Gansu Province, totaling around 38.32 × 10^4^ km^2^. Low‐suitability areas are primarily located in Qinghai Province, Tibet Autonomous Region, Sichuan Province, Yunnan Province, the northern part of Shaanxi Province, some areas in the northeast of Inner Mongolia Autonomous Region, and most parts of Shanxi Province, covering an area of 95.05 × 10^4^ km^2^. Furthermore, other provinces and cities are deemed unsuitable for the distribution of *A. yunnanensis* var. *labrangensis*.

### 3.5 Influencing Factors of Potential Geographical Distribution of *Aster* Species

In the maxent model used to analyze the potential distribution of *A. yunnanensis* var. *labrangensis* under current climate conditions, environmental variables with contribution rates > 5 include mean diurnal range (Bio2, 15.7%), isothermality (Bio3, 27.1%), mean temperature of warmest quarter (Bio10, 21.4%), annual precipitation (Bio12, 26.2%) and precipitation seasonality (coefficient of variation) (Bio15, 7.4%). These variables significantly influence the habitat distribution of *A. yunnanensis* var. *labrangensis* (Table S3). The single‐factor response curve (Figure S15) indicates that the suitable environmental conditions for *A. yunnanensis* var. *labrangensis* are as follows: mean diurnal range (Bio2) of 14.5°C–15.5°C, isothermality (Bio3) range of 38–43, min temperature of coldest month (Bio6) of −23°C to −20°C, mean temperature of warmest quarter (Bio10) of 7°C–12°C, annual precipitation (Bio12) of 500–700 mm, precipitation of driest month (Bio14) of 1–4 mm, and precipitation seasonality. Precipitation seasonality (Bio15) of 86%–98%. All variables exhibit a unimodal response, with peak values at Bio2 = 15°C, Bio3 = 40, Bio10 = 9°C, Bio12 = 510 mm, Bio14 = 3 mm, and Bio15 = 92%. Notably, precipitation in the driest month (Bio14) shows the highest sensitivity, with a sharp decline in presence probability outside the 1–4 mm range. Model predictions reflect environmental preferences within observed data and may not capture unsampled suitable areas.

Key variables influencing its distribution of the *A. diplostephioides* (Table S3) include mean diurnal range (Bio2, 0.2%), isothermality (Bio3, 55.9%), mean temperature of warmest quarter (Bio10, 16.4%), precipitation of driest month (Bio14, 0.2%), precipitation seasonality (coefficient of variation) (Bio15, 1.4%) and precipitation of warmest quarter (Bio18, 25.9%). All variables show unimodal responses (see Figure S10), with isothermality (Bio3) being the most influential, indicating strict requirements for temperature–precipitation combinations during the growing season. High‐probability zones correspond to Bio2 = 12°C–15°C, Bio3 = 38–45, Bio10 = 6°C–10°C, Bio14 = 3–8 mm, Bio15 = 80%–100%, and Bio18 = 300–450 mm, with peak values at midpoints of these ranges.

The study on the potential habitat distribution of *A. farreri* using a maxent model revealed that key environmental factors influencing the prediction model included mean diurnal range (Bio2, 13.2%), isothermality (Bio3, 5.1%), mean temperature of the driest quarter (Bio9, 21.0%), mean temperature of the warmest quarter (Bio10, 33.6%), annual precipitation (Bio12, 11.6%), precipitation of the driest month (Bio14, 8.4%), and precipitation seasonality (coefficient of variation) (Bio15, 7.1%). Response curves for isothermality (Bio3), mean temperature of the warmest quarter (Bio10), annual precipitation (Bio12), and precipitation seasonality (Bio15) show unimodal patterns (Figure S11), with critical thresholds at Bio3 = −7°C, Bio10 = 11°C, Bio12 = 660 mm, and Bio15 = 90%. Mean diurnal range (Bio2) and mean temperature of the driest quarter (Bio9) exhibit linear positive correlations with presence probability, peaking at 15°C and 18.5°C, respectively, before stabilizing. Precipitation in the driest month (Bio14) shows a threshold effect, with the highest probability at 0 mm and a sharp decline with increasing precipitation.

When predicting the potential distribution of *A. souliei* using the MaxEnt model, it was found that environmental variables such as mean diurnal range (Bio2, 2.9%), isothermality (Bio3, 41.5%), temperature seasonality (standard deviation ×100) (Bio4, 23.0%), mean temperature of warmest quarter (Bio10, 13.9%), annual precipitation (Bio12, 17.5%), precipitation of driest month (Bio14, 0.3%), and precipitation seasonality (coefficient of variation) (Bio15, 1.0%) had a high contribution rate (Table S3). These findings suggest that these specific environmental factors play a significant role in influencing the distribution of *A. souliei* . According to the single‐factor response curve, all variables show unimodal responses (Figure S13), peaking at Bio2 = 15.2°C, Bio3 = 45, Bio4 = 600, Bio10 = 8°C, Bio12 = 700 mm, Bio14 = 3 mm, and Bio15 = 95%. With the exception of isotherm and the average temperature in the warmest season, the distribution rate of *A. souliei* experiences a sharp decrease when the average diurnal temperature range, seasonal temperature variation coefficient, annual precipitation, driest month precipitation, and seasonal change of precipitation exceed the critical point. The range of environmental factors for the high distribution rate of *A. souliei* is relatively broad, except for annual precipitation and precipitation in the driest month. This indicates that narrow suitable ranges for annual precipitation and precipitation in the driest month are the primary limiting factors that impact the distribution of *A. souliei* .

The potential distribution of *A. asteroides* was predicted using a maxent model, which identified eight environmental factors with high contribution rates (Table S3). These factors included mean diurnal range (Bio2, 0.3%), isothermality (Bio3, 17.7%), min temperature of coldest month (Bio6, 0.1%), mean temperature of wettest quarter (Bio8, 1.1%), mean temperature of warmest quarter (Bio10, 66.3%), annual precipitation (Bio12, 13.3%), precipitation of driest month (Bio14, 0.6%), and precipitation of coldest quarter (Bio19, 0.6%). The single‐factor response curve illustrated the impact of these factors, with seven showing unimodal responses and isothermality (Bio3) exhibiting an “increase‐then‐stabilize” pattern (Figure S9) Critical thresholds include Bio2 = 14°C, Bio6 = −22°C to −17°C (low contribution, 3%, but ecologically meaningful), Bio8 = 0°C, Bio10 = 2°C, Bio12 = 350 mm, Bio14 = 0 mm, and Bio19 = 0 mm. Deviations in precipitation‐related variables (Bio12, Bio14, Bio19) drastically reduce presence probability, indicating water is the core limiting factor influencing the distribution of *A. asteroides* .

Significant variables (Table S3) include mean diurnal range (Bio2, 6.4%), isothermality (Bio3, 1.4%), temperature seasonality (standard deviation ×100) (Bio4, 31.9%), mean temperature of warmest quarter (Bio10, 26.8%), mean temperature of coldest quarter (Bio11, 14.8%), annual precipitation (Bio12, 13.6%), precipitation of driest month (Bio14, 0.5%), and precipitation seasonality (coefficient of variation) (Bio15, 4.7%). MaxEnt Analysis of the single factor curve (see Figure S12) indicated that the most suitable environmental conditions for the distribution of *A. poliothamnus* are a diurnal temperature range of 15°C–15.5°C, isothermality greater than 40, seasonal temperature variation coefficient of 610–750, average temperature of the warmest quarter 8°C–14.5°C, average temperature of the coldest quarter −7°C to −2°C, annual precipitation of 750 mm, and seasonal variation of precipitation of 90–98 mm. Temperature seasonality (Bio4), mean temperatures of the warmest/coldest quarters (Bio10, Bio11), and annual precipitation (Bio12) show unimodal responses, while mean diurnal range (Bio2) and precipitation seasonality (Bio15) exhibit complex “increase‐steep decline‐stabilize” patterns.

The contribution rate and permutation importance of the main environmental variables in predicting the potential distribution of *A. tongolensis* were analyzed using the MaxEnt model; these variables include a mean diurnal range (Bio2, 5.2%), isothermality (Bio3, 20.2%), temperature seasonality (standard deviation ×100) (Bio4, 27.9%), mean temperature of warmest quarter (Bio10, 9.9%), mean temperature of coldest quarter (Bio11, 15.5%), annual precipitation (Bio12, 19.0%), precipitation seasonality (Coefficient of Variation) (Bio15, 0.8%), and precipitation of coldest quarter (Bio19, 1.4%). The single‐factor response curves (see Figure S14) show that *A. tongolensis* presence probability exhibits unimodal responses to most variables. Suitable conditions for the development of *A. tongolensis* are observed when the average daily temperature range is 11°C–15°C, isotherm is 43–47, seasonal temperature variation coefficient is 550–680, average temperature in the warmest quarter is 7.5°C–14°C, average temperature in the coldest quarter is −5°C–3°C, annual precipitation is 600–900 mm, seasonal variation of precipitation is 86–97 mm, and coldest quarterly precipitation is 10–40 mm. Notably, while annual precipitation (Bio12) and precipitation of coldest quarter (Bio19) act as primary limiting factors with narrow suitability ranges, *A. tongolensis* displays broad tolerance to other variables, indicating low environmental specificity except for moisture‐related parameters.

### 3.6 Changes of Suitable Areas of *Aster* Under Future Climate Conditions

In the prediction of potential distribution of *A. asteroides* under various climate scenarios, it is observed that the suitability area of *A. asteroides* shrinks significantly in Qinghai Province and Sichuan Province (Figure S16). Specifically, under the ssp126 climate scenario, the suitability area of *A. asteroides* has expanded in Tibet Autonomous Region from the current non‐suitability area to the suitability area, but the extent of expansion is less than the extent of contraction. Due to different CO_2_ emission scenarios in the future, some of the suitable areas of *A. asteroides* will become non‐suitable areas, and the shrinkage of the suitable areas will mainly occur in Qinghai Province and Tibet Autonomous Region. By 2040–2060, the suitable area of *A. asteroides* will shrink by 22.78 × 10^4^ km^2^, 22.37 × 10^4^ km^2^, 15.87 × 10^4^ km^2^ and 53.16 × 10^4^ km^2^ under the climate scenarios ssp126, ssp45, ssp370, and ssp585, respectively. In 2080–2100, it will shrink 13.90 × 10^4^ km^2^, 22.56 × 10^4^ km^2^, 41.12 × 10^4^ km^2^ and 25.63 × 10^4^ km^2^, respectively. In 2040–2060, the suitable areas for the expansion of *A. asteroides* are 2.55 × 10^4^ km^2^, 0.98 × 10^4^ km^2^, 2.23 × 10^4^ km^2^ and 0.31 × 10^4^ km^2^, respectively. The expansion area in 2080–2100 will be 4.49 × 10^4^ km^2^, 3.46 × 10^4^ km^2^, 1.04 × 10^4^ km^2^ and 2.01 × 10^4^ km^2^, respectively (Table 2). Overall, regardless of the future climate scenario, the shrinkage area for *A. asteroides* is significantly larger than the expansion area, suggesting a decrease in the distribution of *A. asteroides* in the future climate.

**TABLE 2:** Changes of suitable area of seven species of aster under different climate conditions in the future.

Species	Climate	2040–2060			2080–2100		
		Range expansion (×10^4^ km^2^)	Range contraction (×10^4^ km^2^)	No change (×10^4^ km^2^)	Range expansion (×10^4^ km^2^)	Range contraction (×10^4^ km^2^)	No change (×10^4^ km^2^)
*Aster. asteroides*	ssp126	2.55	22.78	73.77	4.49	13.9	82.64
	ssp245	0.98	22.37	74.18	3.46	22.56	73.98
	ssp370	2.23	15.87	80.67	1.04	41.12	55.43
	ssp585	0.31	53.16	43.38	2.01	25.63	70.92
*Aster diplostephioides*	ssp126	2.17	8.71	44	2.7	6.08	46.64
	ssp245	2.95	8.93	43.78	3.32	6.32	46.39
	ssp370	2.97	8.28	44.43	2.85	13.58	39.13
	ssp585	0.66	16.97	35.74	3.49	9.69	43.02
*Aster farreri*	ssp126	3.84	1.79	38.17	3.11	2.39	37.57
	ssp245	3.86	2.53	37.43	9.58	1.3	38.65
	ssp370	5.44	1.76	38.19	7.09	3	36.95
	ssp585	5.53	2.11	37.84	8.3	1.98	37.97
*Aster poliothamnus*	ssp126	10.04	2.37	34.87	5.54	1.78	35.46
	ssp245	14.53	1.72	35.52	8.66	2.3	34.94
	ssp370	28.98	0.87	36.37	10.31	3.58	33.66
	ssp585	13.99	4.07	33.17	11.68	1.74	35.5
*Aster souliei*	ssp126	7.13	2.18	33.41	3.13	5.12	30.47
	ssp245	5.8	2.21	33.38	9.67	2.52	33.07
	ssp370	7.56	2.1	33.49	4.85	6.61	28.98
	ssp585	6.99	3.06	32.53	5.52	3.8	31.79
*Aster tongolensis*	ssp126	3.67	2.09	31.64	1.11	3.11	30.62
	ssp245	6.6	1.83	31.9	3.35	2.02	31.71
	ssp370	6.78	2.06	31.68	4.91	2.8	30.94
	ssp585	5.76	1.13	32.61	9.43	1.38	32.36
*Aster yunnanensis* var. *labrangensis*	ssp126	3.21	0.88	34.17	4.21	1.22	33.83
	ssp245	1.8	2.73	32.31	3.93	4.5	30.55
	ssp370	3.17	3.4	31.65	2.52	2.45	32.59
	ssp585	2.2	3.84	31.21	5.69	0.82	34.22

In the climate scenarios ssp126, ssp245, ssp370, and ssp585 from 2040 to 2060, the suitable area of *A. diplostephioides* is projected to decrease by 8.71 × 10^4^ km^2^, 8.93 × 10^4^ km^2^, 8.28 × 10^4^ km^2^, and 16.97 × 10^4^ km^2^, respectively, compared to current conditions (Figure S17). Conversely, non‐suitable areas are expected to expand into suitable areas by 2.17 × 10^4^ km^2^, 2.95 × 10^4^ km^2^, 2.97 × 10^4^ km^2^, and 0.66 × 10^4^ km^2^ in the same period. Moving forward to the period from 2080 to 2100 under the same climate scenarios, the suitable area of *A. diplostephioides* is forecasted to shrink by 6.08 × 10^4^ km^2^, 6.32 × 10^4^ km^2^, 13.58 × 10^4^ km^2^, and 9.69 × 10^4^ km^2^, respectively, in comparison to current climate conditions. Concurrently, non‐suitable areas are projected to expand into suitable areas by 2.70 × 10^4^ km^2^, 3.32 × 10^4^ km^2^, 2.85 × 10^4^ km^2^, and 3.49 × 10^4^ km^2^, respectively (Table 2). The reduction in suitable area is anticipated to primarily affect Yunnan Province and the Tibet Autonomous Region, with a minor expansion area located in the Tibet Autonomous Region and Qinghai Province. Overall, *A. diplostephioides* is expected to experience less expansion than contraction under different climate scenarios in both the 2040–2060 and 2080–2100 periods, suggesting that future climate conditions may not be conducive to the growth of this plant species.

In the projected climate scenarios of 2040–2060 (ssp126, ssp245, ssp370, and ssp585), the suitable area for *A. farreri* is estimated to expand by 3.84 × 10^4^ km^2^, 3.86 × 10^4^ km^2^, 5.44 × 10^4^ km^2^, and 5.53 × 10^4^ km^2^, respectively, compared to current conditions (Figure S18). Conversely, the areas transitioning from suitable to non‐suitable are 1.79 × 10^4^ km^2^, 2.53 × 10^4^ km^2^, 1.76 × 10^4^ km^2^, and 2.11 × 10^4^ km^2^ under the respective scenarios. Looking ahead to 2080–2100, the projected expansion of suitable areas under the same scenarios will be 3.11 × 10^4^ km^2^, 9.58 × 10^4^ km^2^, 7.09 × 10^4^ km^2^, and 8.30 × 10^4^ km^2^, with transitions from suitable to non‐suitable areas being 2.39 × 10^4^ km^2^, 1.30 × 10^4^ km^2^, 3.00 × 10^4^ km^2^, and 1.98 × 10^4^ km^2^, respectively (Table 2). This suggests a trend of overall expansion in suitable areas for *A. farreri* under future climate scenarios, particularly near the Ganzi Tibetan Autonomous Prefecture in Sichuan Province, while the unsuitable areas for *A. farreri* are diminishing. The four future emission models are expected to be conducive to the growth of *A. farreri* .

In the future climate scenarios, there is a noticeable trend towards the expansion of the suitable area for *A. poliothamnus* (Figure S19). Projections under emission scenarios ssp126, ssp245, ssp370, and ssp585 indicate that in 2040–2060, the suitable area of *A. poliothamnus* will expand by 10.04 × 10^4^ km^2^, 14.53 × 10^4^ km^2^, 28.98 × 10^4^ km^2^, and 13.99 × 10^4^ km^2^, respectively. In 2080–2100, the expansion is expected to be 5.54 × 10^4^ km^2^, 8.66 × 10^4^ km^2^, 10.31 × 10^4^ km^2^, and 11.68 × 10^4^ km^2^, respectively; this expansion primarily takes place in Gansu Province, Tibet Autonomous Region, Qinghai Province, and Sichuan Province. Notably, under the ssp370 emission scenario, the expansion of *A. poliothamnus* is most significant by 2040–2060. The areas transitioning from current suitable to non‐suitable for *A. poliothamnus* are projected to be 2.37 × 10^4^ km^2^, 1.72 × 10^4^ km^2^, 0.87 × 10^4^ km^2^, and 4.07 × 10^4^ km^2^ by 2040–2060, with a subsequent shrinkage of 1.78 × 10^4^ km^2^, 2.30 × 10^4^ km^2^, 3.58 × 10^4^ km^2^, and 1.74 × 10^4^ km^2^ by 2080–2100 (Table 2). Overall, the expansion of suitable areas outweighs the contraction, suggesting favorable future climate conditions for *A. poliothamnus* growth and distribution.

In the future years 2040–2060, the suitable area for *A. souliei* will slightly decrease compared to current climate conditions (Figure S20). Across climate scenarios ssp126, ssp245, ssp370, and ssp585, the suitable growing area of *A. souliei* is projected to decrease by 2.18 × 10^4^ km^2^, 2.21 × 10^4^ km^2^, 2.10 × 10^4^ km^2^, and 3.06 × 10^4^ km^2^, respectively. From 2080 to 2100, the suitable area is expected to shrink by 5.12 × 10^4^ km^2^, 2.52 × 10^4^ km^2^, 6.61 × 10^4^ km^2^, and 3.80 × 10^4^ km^2^, respectively. At the same time, some unsuitable areas of *A. souliei* are being extended to suitable areas due to changing climatic conditions. In the 2040–2060 period, the suitable area under the ssp126, ssp245, ssp370, and ssp585 climate scenarios is estimated to be 7.13 × 10^4^ km^2^, 5.80 × 10^4^ km^2^, 7.56 × 10^4^ km^2^, and 6.99 × 10^4^ km^2^, respectively. By 2080–2100, it is projected to expand by 3.13 × 10^4^ km^2^, 9.67 × 10^4^ km^2^, 4.85 × 10^4^ km^2^, and 5.52 × 10^4^ km^2^, respectively (Table 2). Overall, regardless of the climate emission scenario, the expansion area of the suitable *A. souliei* habitat in 2040–2060 surpasses the contraction area, with the expansion primarily in Sichuan Province, Tibet Autonomous Region, Yunnan Province, and Qinghai Province. Under the ssp126 and ssp370 climate scenarios from 2080 to 2100, the shrinking suitable area outweighs the expanding area, indicating a negative impact on the growth of *A. souliei* over time. However, under the ssp245 and ssp585 emission models, the expansion area of suitable habitat exceeds the contraction area, particularly under the ssp245 scenario where the expansion area in 2080–2100 surpasses that of 2040–2060, mainly in Sichuan Province, Yunnan Province, and Tibet Autonomous Region.

Under future climate conditions, the suitable areas of *A. tongolensis* are expected to remain relatively stable, with only minor expansions and contractions (Figure S21). In 2040–2060, the expansion areas for the four climate scenarios (ssp126, ssp245, ssp370, ssp585) are projected to be 3.67 × 10^4^ km^2^, 6.60 × 10^4^ km^2^, 6.78 × 10^4^ km^2^, and 5.76 × 10^4^ km^2^, respectively. By 2080–2100, the expansion areas are estimated to be 1.11 × 10^4^ km^2^, 3.35 × 10^4^ km^2^, 4.91 × 10^4^ km^2^, and 9.43 × 10^4^ km^2^, respectively. Conversely, the shrinking areas during 2040–2060 are anticipated to be 2.09 × 10^4^ km^2^, 1.83 × 10^4^ km^2^, 2.06 × 10^4^ km^2^, and 1.13 × 10^4^ km^2^, while during 2080–2100, they are predicted to be 3.11 × 10^4^ km^2^, 2.02 × 10^4^ km^2^, 2.80 × 10^4^ km^2^, and 1.38 × 10^4^ km^2^. Except for the ssp126 scenario from 2040 to 2060, where the shrinking area of *A. tongolensis* is less than the expansion area, in other scenarios and timeframes, the expansion of *A. tongolensis* exceeds the contraction, albeit not significantly. These expansions are mainly concentrated in Qinghai Province, Tibet Autonomous Region, and Sichuan Province, while contractions are primarily observed in Yunnan Province and Tibet Autonomous Region. Overall, these results indicate that the suitable habitat range of *A. tongolensis* is projected to expand slightly in the future across various climate scenarios, and the future climatic conditions are expected to be favorable for the growth and development of *A. tongolensis* .

Through modeling the potential distribution of *A. yunnanensis* var. *labrangensis* under four future climate scenarios (ssp126, ssp245, ssp370, and ssp585) for the periods 2040–2060 and 2080–2100, significant range dynamics were projected (see Figure S22); the findings indicate that, compared to the current climate conditions, *A. yunnanensis* var. *labrangensis* is projected to expand its suitable area by 3.21 × 10^4^ km^2^, 1.80 × 10^4^ km^2^, 3.17 × 10^4^ km^2^, and 2.20 × 10^4^ km^2^ during the period of 2040–2060 under ssp126, ssp245, ssp370, and ssp585 scenarios, respectively. The stable suitable areas are measured at 34.17 × 10^4^ km^2^, 32.31 × 10^4^ km^2^ × 10^4^ km^2^, 31.65 × 10^4^ km^2^, and 31.21 × 10^4^ km^2^. Conversely, the shrinking areas of the suitable regions were found to be 0.88 × 10^4^ km^2^, 2.73 × 10^4^ km^2^, 3.40 × 10^4^ km^2^, and 3.84 × 10^4^ km^2^ under the same scenarios. In 2080–2100, under ssp126, ssp245, ssp370, and ssp585 climate scenarios, the suitable area of *A. yunnanensis* var. *labrangensis* is projected to decrease by 1.22 × 10^4^ km^2^, 4.50 × 10^4^ km^2^, 2.45 × 10^4^ km^2^, and 0.82 × 10^4^ km^2^, respectively. The suitable area of *A. yunnanensis* var. *labrangensis* has expanded by 4.21 × 10^4^ km^2^, 3.93 × 10^4^ km^2^, 2.52 × 10^4^ km^2^, and 5.69 × 10^4^ km^2^ when compared to the current climate situation (Table 2). In summary, the suitable area of *A. yunnanensis* var. *labrangensis* shows an increase under ssp126 climate scenarios from 2040 to 2060, while it decreases under ssp245, ssp370, and ssp585 climate models. Notably, under ssp126, ssp370, and ssp585 climate scenarios, the suitable area of *A. yunnanensis* var. *labrangensis* is projected to increase in the 2080–2100 period, whereas a decline is predicted under the ssp245 scenario. The reduction in the suitable area of *A. yunnanensis* var. *labrangensis* is expected to mainly occur in Sichuan Province and Yunnan Province, while expansion is anticipated in Qinghai Province and the Tibet Autonomous Region.

### 3.7 Centroid Transfer of *Aster* in Suitable Areas Under Different Climate Scenarios

**FIGURE 4:**
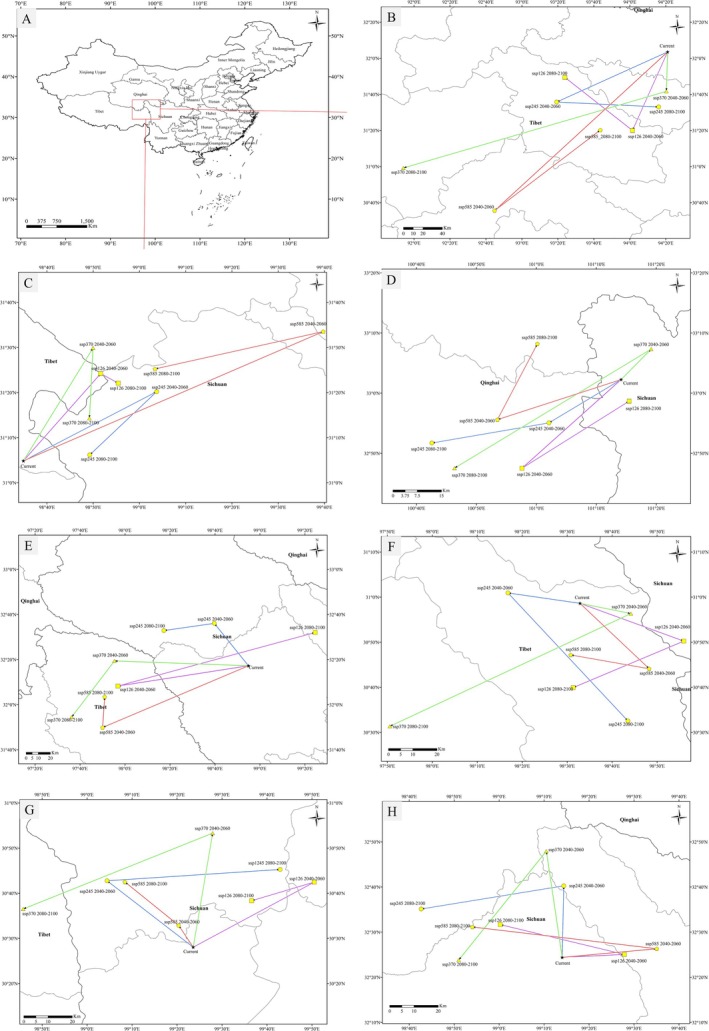
Centroid transfer of seven species of *Aster* under different climatic conditions in the future. (A) *Aster* plant distribution center, (B) *Aster asteroides* , (C) *Aster diplostephioides* , (D) *Aster farreri* , (E) *Aster poliothamnus*, (F) *Aster souliei*, (G) *Aster tongolensis* , and (H) *Aster yunnanensis* var. *labrangensis*.

The current distribution center of *A. asteroides* is located in Baqing County, Nagqu City, Tibet Autonomous Region (Figure 4B). By 2040–2060, the distribution center is projected to shift to Biru County, Nagqu City under climate scenarios ssp126, ssp245, and ssp370. In the ssp585 climate scenario, the distribution center is expected to move to Jiali County of Nagqu City during the same time period. By 2080–2100, under ssp126 and ssp585 climate scenarios, the distribution centers will revert back to Bilu County, while under ssp245 and ssp370 climate scenarios, the centers will shift to Suo County, Nagqu City. This suggests that the distribution center of knotweed will move southwestward under varying climate scenarios in the future, but it will remain within Nagqu City, Tibet Autonomous Region.

The current distribution center of *A. diplostephioides* is located in Jiangda County, Changdu City, Tibet Autonomous Region (Figure 4C). However, under future climate conditions, the distribution of *A. diplostephioides* is projected to shift northeastward. From 2040–2060 to 2080–2100, the distribution center of *A. diplostephioides* will undergo changes based on different climate scenarios: ssp126—Jiangda County to Baiyu County, ssp245—Baiyu County to Baiyu County, ssp370—Dege County to Baiyu County, and ssp585—Ganzi County to Baiyu County. This suggests that regardless of the climate emission model, the distribution center of *A. diplostephioides* is expected to shift to Baiyu County, Garze Tibetan Autonomous Prefecture, Sichuan Province by 2080–2100.

The distribution center of *A. farreri* is currently located in Aba County, Aba Tibetan Autonomous Prefecture, Sichuan Province (Figure 4D). Depending on the climate scenario, the distribution center will shift over time. Under the ssp126 scenario, it will move from Aba County to Banma County, Guoluo Tibetan Autonomous Prefecture, Qinghai Province, and then back to Aba County. In the ssp245 scenario, the distribution center remains the same from Aba County to Banma County. For the ssp370 scenario, the distribution center will remain in Aba County until 2040–2060 and then shift to Banma County by 2080–2100. Lastly, in the ssp585 scenario, the distribution center will move from Aba County to Banma County and then to Jiuzhi County in Guoluo Tibetan Autonomous Prefecture, Qinghai Province.

The distribution center of *A. poliothamnus* is currently located in Dege County, Garze Tibetan Autonomous Prefecture, Sichuan Province (Figure 4E). It is projected to shift westward in the future. By 2040–2060, under ssp126, ssp370, and ssp585 climate scenarios, the distribution center of *A. poliothamnus* will move to Jiangda County, Tibet Autonomous Region. For the ssp245 climate scenario, the distribution center of *A. poliothamnus* will shift to Shiqu County, Garze Tibetan Autonomous Prefecture, and remain there from 2080 to 2100. As for the ssp585 climate scenario, the distribution center of *A. poliothamnus* will relocate from Jiangda County, Tibet Autonomous Region, to Ganzi County, Sichuan Province, by 2080–2100. However, under the ssp370 and ssp585 climate scenarios, the distribution center of *A. poliothamnus* will still be in Jiangda County.

The distribution center of *A. souliei* is currently located in Gongjue County, Changdu City, Tibet Autonomous Region (Figure 4F). In future climate scenarios ssp126 and ssp585, the distribution center will continue to be in Gongjue County. However, under the ssp245 scenario, the distribution center will temporarily shift to Baiyu County between 2040 and 2060, before returning to Gongjue County from 2080 to 2100. For the ssp370 scenario, the distribution center will remain in Gongjue County from 2040 to 2060, but will move to Chaya County, Qamdo City, Tibet Autonomous Region from 2080 to 2100. Ultimately, in 2080–2100, the distribution center of *A. souliei* is projected to be in Qamdo City within the Tibet Autonomous Region.

The distribution center of *A. tongolensis* is currently located in Batang County, Garze Tibetan Autonomous Prefecture, Sichuan Province, but is projected to move northward in the future (Figure 4G). Between 2040 and 2060, under the ssp245, ssp370, and ssp585 climate scenarios, the distribution center of *A. tongolensis* will shift to Baiyu County. For the ssp245 and ssp585 scenarios, it will remain in Baiyu County by 2080–2100, while for the ssp370 scenario, it will move to Gongjue County, Changdo City, and Tibet Autonomous Region during the same period. In the ssp126 scenario, the distribution center of *A. tongolensis* will shift from Batang County to Xinlong County and then to Baiyu County, indicating that Garze Tibetan Autonomous Prefecture, Sichuan Province, will be the main location for the distribution center of *A. tongolensis* .

The distribution center of *A. yunnanensis* var. *labrangensis* (Figure 4H), currently located in Dege County, Garze Tibetan Autonomous Prefecture, Sichuan Province, is projected to shift under different climate scenarios. In the ssp126 and ssp370 scenarios, the center will move northward to Ganze County between 2040 and 2060 and then westward to Dege County by 2080–2100. Alternatively, under the ssp245 and ssp585 scenarios, the center will shift from Ganzi County to Shiqu County between 2040 and 2060 and continue westward from 2080 to 2100.

## 4 Discussion

Based on the data from multiple sources including the Flora of China, Flora of Qinghai, Flora of Tibet, Digital Herbarium of China, and the Global Biodiversity Information Facility (GBIF) (Lin and Chen 1985; Liu 1996; Shi, Zhu, and Wang 2023), and supplemented by field investigations, we compiled distribution records for seven *Aster* species across China. Occurrence data were geographically filtered to retain only high‐quality, spatially unique records to ensure model robustness. While these species are predominantly distributed in the Qinghai‐Tibet Plateau (> 95% of records), isolated populations of *A. farreri* , *A. tongolensis* , and *A. poliothamnus* were documented in other mountainous regions of China, such as Xiaowutai Mountain (Hebei Province) and Wutai Mountain (Shanxi Province) for *A. farreri* .

To assess the broader distribution potential of *A. farreri* beyond the plateau, we applied the MaxEnt model using seven carefully selected environmental variables (Table S3), including annual precipitation (Bio12), mean temperature of the warmest quarter (Bio10), and precipitation seasonality (Bio15). These variables were selected based on their ecological relevance to alpine plant physiology, with highly correlated predictors (|*r*| > 0.8) excluded. The model revealed that climatically suitable areas for *A. farreri* extend beyond the Qinghai‐Tibet Plateau, particularly in mountainous regions with comparable water and temperature regimes. The broad distribution of *A. farreri* likely reflects its physiological plasticity to temperature gradients (Bio10 contribution = 33.6%), enabling colonization of both high‐altitude plateaus and mid‐elevation mountains. In contrast, narrowly distributed species such as *A. souliei* rely heavily on stable isothermality (Bio3 contribution = 41.5%), rendering them more vulnerable to climate warming. This aligns with field observations of isolated populations in northern China, suggesting *A. farreri* may tolerate broader environmental conditions than previously recognized. However, it is critical to note that the model's training data were constrained to Chinese territory, potentially underrepresenting the full ecological niche if suitable habitats exist beyond national borders. Based on projected range changes, we recommend: (1) Core habitat protection: Establish nature reserves in stable suitable areas of *A. souliei* (Qinghai‐Tibet Plateau). (2) Migration corridor facilitation: Maintain connectivity between current and future suitable areas for *A. farreri* . (3) Adaptive management: Prioritize ex situ conservation for species with shrinking habitats in future ssp. scenarios ( *A. asteroides* , Table 2).

*A. poliothamnus* typically occurs on slopes and banks of streams at altitudes of 1800–3300 m. This wild species has been documented in the Global Biodiversity Information Facility (GBIF) (https://www.gbif.org/zh/) in eastern China and the Qinghai‐Tibet Plateau. Based on distribution data, this study predicts a limited suitable habitat range of *A. poliothamnus* in southeastern China, excluding coastal areas. These GBIF points in eastern China (inland provinces) were included in the model, but potential coastal records were excluded due to suspected cultivation or coordinate errors. This discrepancy may be attributed to the sparse natural distribution of *A. poliothamnus* in these areas, potentially due to insufficient field verification, artificial cultivation (non‐naturalized), or model limitations (e.g., overfitting, limited environmental variables). Model predictions indicate that central‐eastern China, excluding the Qinghai‐Tibet Plateau, has suitable hydrothermal conditions (Bio10: 9°C–12°C, Bio12: 800–1000 mm/year) for *A. poliothamnus*. However, modeled suitability in coastal provinces remains low, possibly due to unmodeled factors such as soil acidity or urbanization (Figure S5). Future efforts could focus on artificial cultivation in central‐eastern China to enhance genetic resource utilization, while verifying naturalization potential in coastal zones.

This study employed a MaxEnt model and six to eight climate factors related to temperature and precipitation to forecast the potential suitable distribution area of *Aster* species in the source region of the Yellow River. It also explored the changes in the suitable area and the shift of distribution centers under various climate scenarios in the future. Model‐predicted AUC values for seven species of *Aster* exceeded 0.9, indicating a high level of prediction accuracy. The study successfully predicted the potential distribution of seven species of *Aster* in China under both current and future climate scenarios (Guisan and Thuiller 2005; An et al. 2023). During the climate model establishment, significant environmental factors influencing *Aster* species were identified based on distribution points and climate factors. The analysis revealed that temperature and precipitation were the predominant environmental factors limiting the potential distribution of *Aster*. Six to eight important climate factors were identified, including mean diurnal range (Bio2), isothermality (Bio3), and mean temperature of warmest quarter (Bio10), which were common among the seven *Aster* species, similar to medicinal *orchids* in Darjeeling eastern Himalaya (Boral and Moktan 2024). Annual precipitation (Bio12) emerged as a primary driver (contribution: 22%–35%; Table S2), included for six *Aster* species except *A. diplostephioides*, while precipitation of the driest month (Bio14, used in six species, contribution: 0.1%–9%) and coldest quarter (Bio19, used only in two species, contribution: 0.1%–9%) exhibited modest but ecologically relevant effects on *Aster* distribution. These variables likely influence water stress tolerance and overwintering survival, particularly in arid alpine regions. This is in line with previous findings suggesting that precipitation of the driest month (Bio14) and precipitation of the wettest month (Bio13) strongly influence the suitable areas of *Symphyotrichum subulatum* and *Ephedra* (Li et al. 2023; He et al. 2021), highlighting the substantial impact of precipitation on the distribution of *Aster* species.

Under the current climate, the seven species of *Aster* are mainly located in the southeastern area of the Qinghai‐Tibet Plateau, encompassing Guoluo Tibetan Autonomous Prefecture and Yushu Tibetan Autonomous Prefecture in Qinghai Province, Qamdo City in Tibet Autonomous Region, Garze Tibetan Autonomous Prefecture and Aba Tibetan Autonomous Prefecture in Sichuan Province, Diqing Tibetan Autonomous Prefecture in Yunnan Province, and Gannan Tibetan Autonomous Prefecture in Gansu Province. The *Aster* species with the largest suitable area is the widely distributed *A. farreri* , covering 341.99 × 10^4^ km^2^. It is followed by *A. asteroides* and *A. poliothamnus*, with suitable areas of 261.36 × 10^4^ km^2^ and 247.71 × 10^4^ km^2^, respectively, based on the 10th percentile training presence threshold (0.2117, 0.3969, and 0.2605 for each species; Table S4). The remaining four species of *Aster* have suitable areas less than 200 × 10^4^ km^2^, with *A. souliei* having the smallest suitable area at 139.66 × 10^4^ km^2^. The medium and low suitability areas of *A. farreri* are notably larger compared to other species. *A. asteroides* stands out for having the highest area of high suitability at 74.85 × 10^4^ km^2^, significantly greater than the other six species. On the other hand, *A. souliei* has the smallest areas of medium and low suitability at 38.23 × 10^4^ km^2^ and 65.86 × 10^4^ km^2^, respectively. The minimum area of high suitability for *A. tongolensis* is 30.13 × 10^4^ km^2^.

In future climate conditions, the suitable habitat for *A. diplostephioides* and *A. asteroides* is projected to decrease rather than expand. Specifically, during 2040–2060 under the ssp585 climate scenario, *A. diplostephioides* and *A. asteroides* are anticipated to experience the largest habitat shrinkage, measuring 16.96 × 10^4^ km^2^ and 52.86 × 10^4^ km^2^, respectively. In contrast, from 2080 to 2100 under the ssp370 climate scenario, the shrinkage area of *A. diplostephioides* and *A. asteroides* is predicted to be highest, with measurements of 10.73 × 10^4^ km^2^ and 40.07 × 10^4^ km^2^, respectively. Importantly, the shrinkage area of *A. asteroides* is forecasted to be larger than that of *A. diplostephioides* . The suitable habitat for *A. diplostephioides* is shrinking in the south and west of its current range due to climate change, while the suitable habitat for *A. asteroides* is decreasing in the east and north. Overall, future climate conditions are not conducive to the growth of *A. diplostephioides* and *A. asteroides* , resulting in a reduction in the habitable area and a shift in the distribution center towards the northwest. This trend is similar to the changes observed in *Larix* species and medicinal plants (such as *Lomatogoniopsis alpina*, *Rheum tanguticum*, *Arenaria brevipetala*, and *Fritillaria*) from the Qinghai‐Tibet Plateau, which are also expected to encounter decreased suitable habitat and high‐altitude migration towards the north in response to future climate conditions (An et al. 2023; Yang et al. 2024). This indicates that these species are more sensitive to climate change. In contrast, suitable habitats for species such as *A. asteroides* may expand, which may be related to rising temperatures on the Qinghai‐Tibet Plateau promoting plant growth, development, and spread. However, it is important to note that while suitable habitats will expand in the future for some species, the area of expansion is likely to be smaller in the 2080–2100 period than in the 2040–2060 period, suggesting that continued global warming may have a negative impact on plant distribution.

During the period from 2040 to 2060, with the exception of ssp126, the suitable area for *A. yunnanensis* var. *labrangensis* is projected to diminish in size compared to the expansion area. Conversely, during the period from 2080 to 2100, except for ssp245, the expansion area is anticipated to exceed the shrinkage area under the other three climate scenarios. However, across different years and climate conditions, no significant disparity is observed in the expansion and contraction areas of *A. yunnanensis* var. *labrangensis*, suggesting that future climatic conditions may not exert a pronounced influence on its growth. On the contrary, the suitable habitats for *A. farreri* , *A. souliei* , *A. poliothamnus*, and *A. tongolensis* are projected to expand under future climate conditions, with *A. poliothamnus* and *A. tongolensis* demonstrating the largest expansion areas. This trend is consistent with similar changes observed in *Symphyotrichum subulatum* , *Fritillaria unibracteata*, *Dracocephalum tanguticum*, and *Dracocephalum heterophyllum* (Li et al. 2023; An et al. 2023; Shi, Zhu, and Wang 2023). The anticipated temperature rise on the Qinghai‐Tibet Plateau is expected to enhance plant growth, development, and dissemination to a certain extent. Consequently, the distribution ranges of *A. yunnanensis* var. *labrangensis*, *A. farreri* , *A. souliei* , *A. poliothamnus*, and *A. tongolensis* are forecast to significantly expand under future climate conditions. Notably, the projected expansion of habitable areas from 2080 to 2100 is smaller than that from 2040 to 2060, indicating that the ongoing temperature increase will affect plant distribution. The adaptability of the seven *Aster* species in the Qinghai‐Tibet Plateau to future climate conditions is ranked as follows: *A. poliothamnus* > *A. souliei* > *A. tongolensis* > *A. farreri* > *A. yunnanensis* var. *labrangensis* > *A. diplostephioides* > *A. asteroides* .

## 5 Conclusion

The potential distribution of seven species of aster on the Qinghai–Tibet Plateau in China was predicted using a maxent model, achieving high model accuracy (AUC > 0.9). In the current climate, the suitable areas for *A. yunnanensis* var. *labrangensis*, *A. diplostephioides* , *A. farreri* , *A. souliei* , *A. asteroides* , *A. poliothamnus*, and *A. tongolensis* in China were determined to be 165.96 × 10^4^ km^2^, 155.38 × 10^4^ km^2^, 341.99 × 10^4^ km^2^, 139.66 × 10^4^ km^2^, 261.36 × 10^4^ km^2^, 247.71 × 10^4^ km^2^, and 177.75 × 10^4^ km^2^, respectively.

Under current climate conditions, the potential suitable areas for the seven species of *Aster* are mainly located on the Qinghai‐Tibet Plateau. The distribution drivers vary among species, with key environmental factors identified through MaxEnt's Jackknife tests and contribution analyses (Table S3). For most species, predictors include isothermality (Bio3), temperature seasonality (standard deviation × 100) (Bio4), mean temperature of the driest quarter (Bio9), mean temperature of the warmest quarter (Bio10), annual precipitation (Bio12), and precipitation of the warmest quarter (Bio18). However, variable importance differs significantly across taxa: isothermality (Bio3) has the highest contribution rate to predicting the distribution of *A. yunnanensis* var. *labrangensis*, *A. diplostephioides* , and *A. souliei* . The mean temperature of the warmest quarter (Bio10) has the highest contribution rate to predicting the distribution of *A. farreri* and *A. asteroides* . The temperature seasonality (Bio4) is the most important environmental factor contributing to the distribution of *A. poliothamnus* and *A. tongolensis* . This revision aligns with best practices in species distribution modeling, where predictor sets are tailored to each taxon's niche requirements rather than applied uniformly.

Under future climate conditions, the suitable areas for *A. diplostephioides* and *A. asteroides* are projected to decrease, while the suitable areas for *A. yunnanensis* var. *labrangensis*, *A. farreri* , *A. souliei* , *A. poliothamnus*, and *A. tongolensis* are expected to increase. By 2100, the distribution centers of *A. yunnanensis* var. *labrangensis* and *A. poliothamnus* will shift westward, while the distribution centers of *A. farreri* , *A. souliei* , and *A. asteroides* will shift toward the southwest. Additionally, the distribution centers of *A. diplostephioides* and *A. tongolensis* will shift to the northeast and north. Despite these shifts, the distribution centers of all seven species of *Aster* are anticipated to remain within the Qinghai‐Tibet Plateau.

To date, the seven species of *Aster* examined in this study have not been utilized for artificial cultivation or introduction and domestication. In the future, based on the predicted outcomes of various aster species from this research, efforts to enhance artificial cultivation and introduction and domestication could be intensified; thereby facilitating the rational development and utilization of these high‐value *Aster* species.

## Author Contributions

**Jinping Qin:** conceptualization (lead), data curation (lead), investigation (lead), resources (equal), software (equal), writing – original draft (equal). **Yanlong Wang:** formal analysis (supporting), investigation (supporting). **Xiaoli Wang:** formal analysis (equal), methodology (equal). **Yuan Ma:** data curation (equal). **Ying Liu:** conceptualization (equal), methodology (equal). **Yushou Ma:** funding acquisition (equal), project administration (equal), resources (equal), supervision (equal), visualization (equal), writing – review and editing (equal).

## Conflicts of Interest

The authors declare no conflicts of interest.

## Supporting information


**Data S1:** ece371931‐sup‐0001‐DataS1.docx.

**Data S2:** ece371931‐sup‐0002‐DataS2.docx.

## Data Availability

Data supporting the findings of this study are publicly available in Supporting Information. The data that support the findings of this study are openly available in Dryad at https://doi.org/10.5061/dryad.gf1vhhn08.
