# Supplementary material for: Prediction of Potential Distribution of Seven Plant Species of Aster (Asteraceae) Based on MaxEnt Model
Source: Ecol Evol. 2025 Sep 30;15(10):e71931. doi: 10.1002/ece3.71931 (PMC12483838; doi:10.1002/ece3.71931)
Supplement: Supplementary file 1 — Data S1: ece371931‐sup‐0001‐DataS1.docx. [file ECE3-15-e71931-s002.docx]

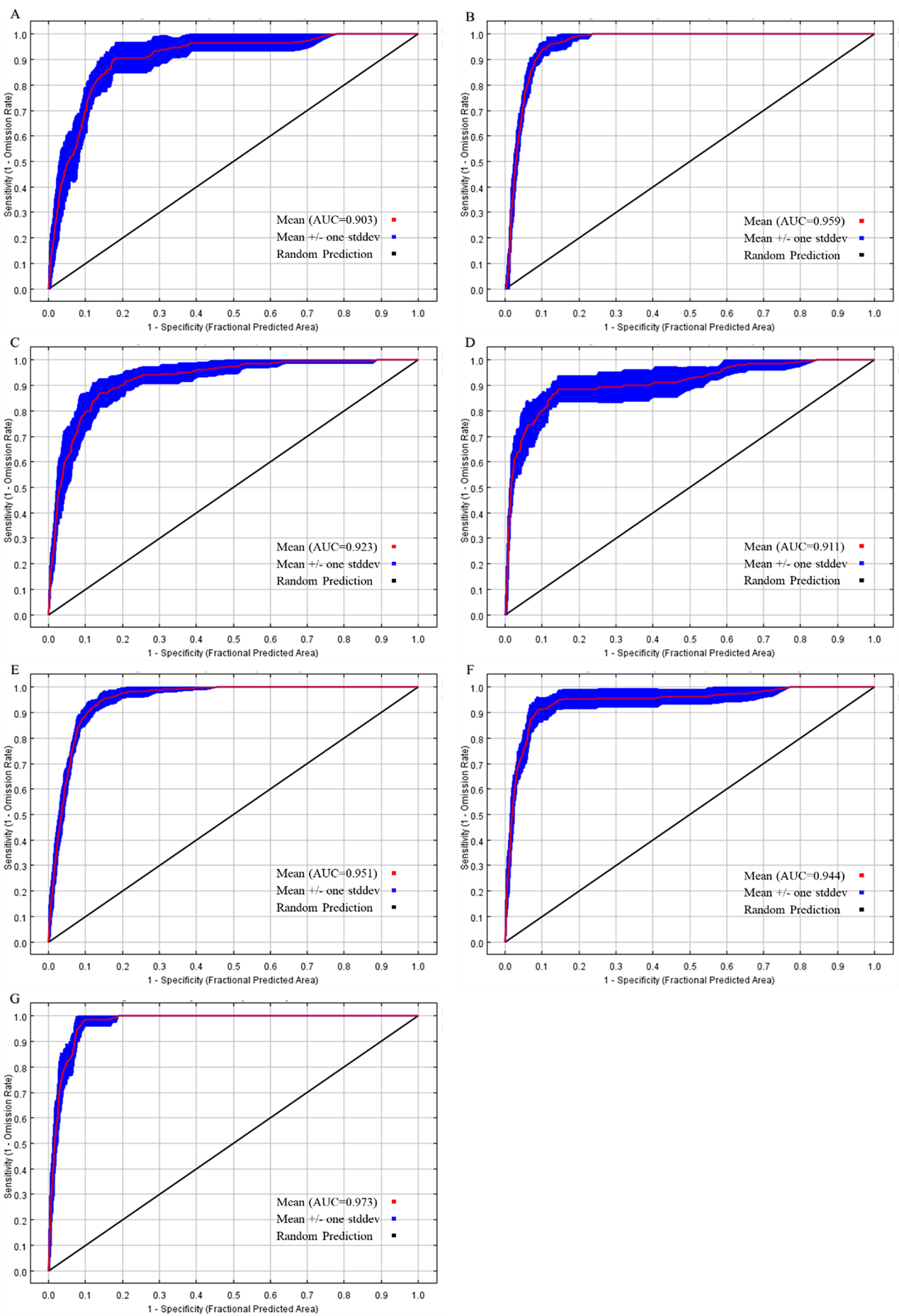


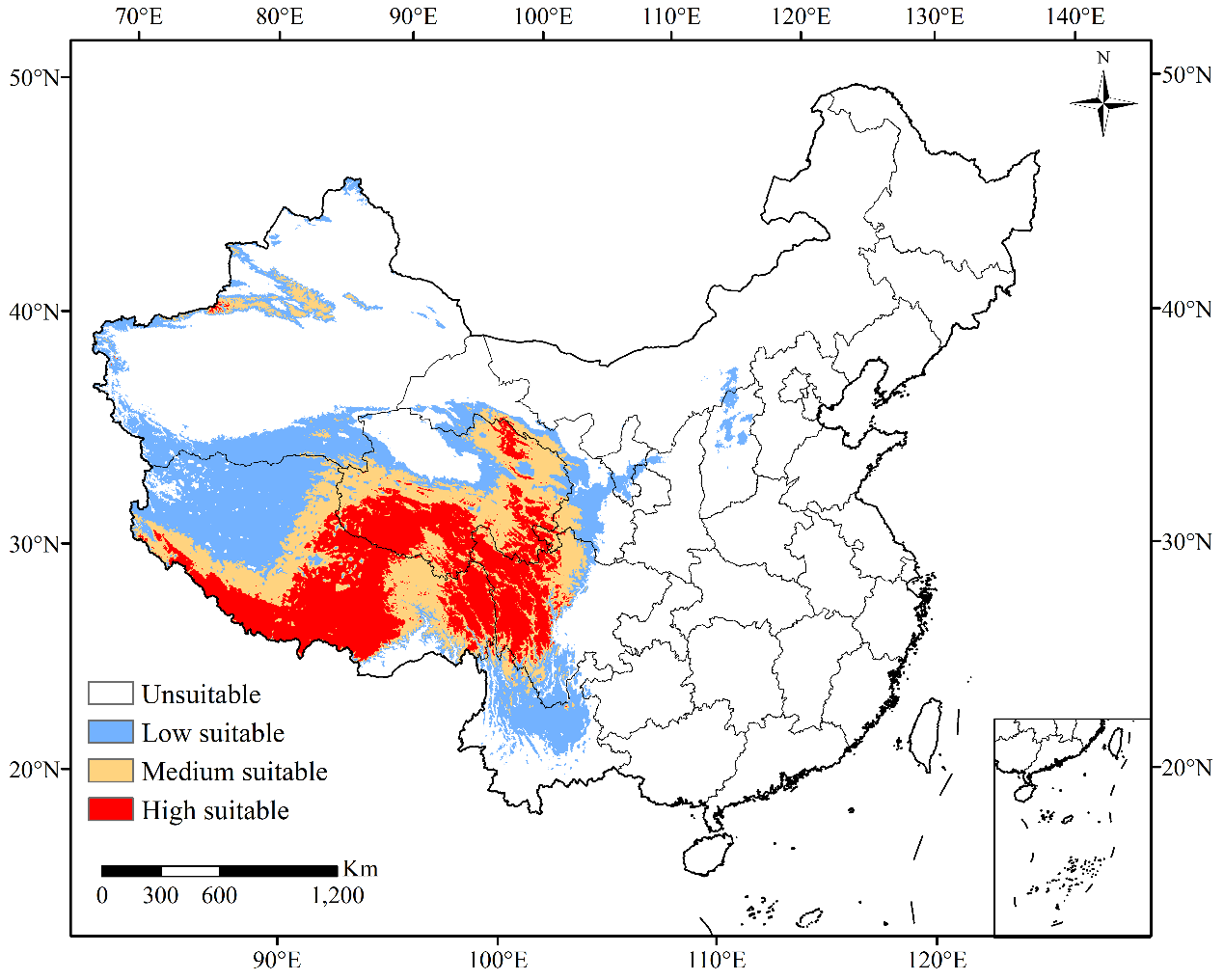


Fig.S1 ROC curve verification of prediction for *Aster* species by MaEent model

A: *Aster asteroides*, B: *Aster diplostephioides*, C: *Aster farreri*, D: *Aster poliothamnus*, E: *Aster souliei*, F: *Aster tongolensis*, and G: *Aster yunnanensis* var*. labrangensis*.

Fig.S2 Potential distribution of *Aster asteroides* in China based on Maxent under current climate.

Note: White: unsuitable area; Blue: low suitability area; Orange: medium suitable area; Red: High suitability area. The same below.


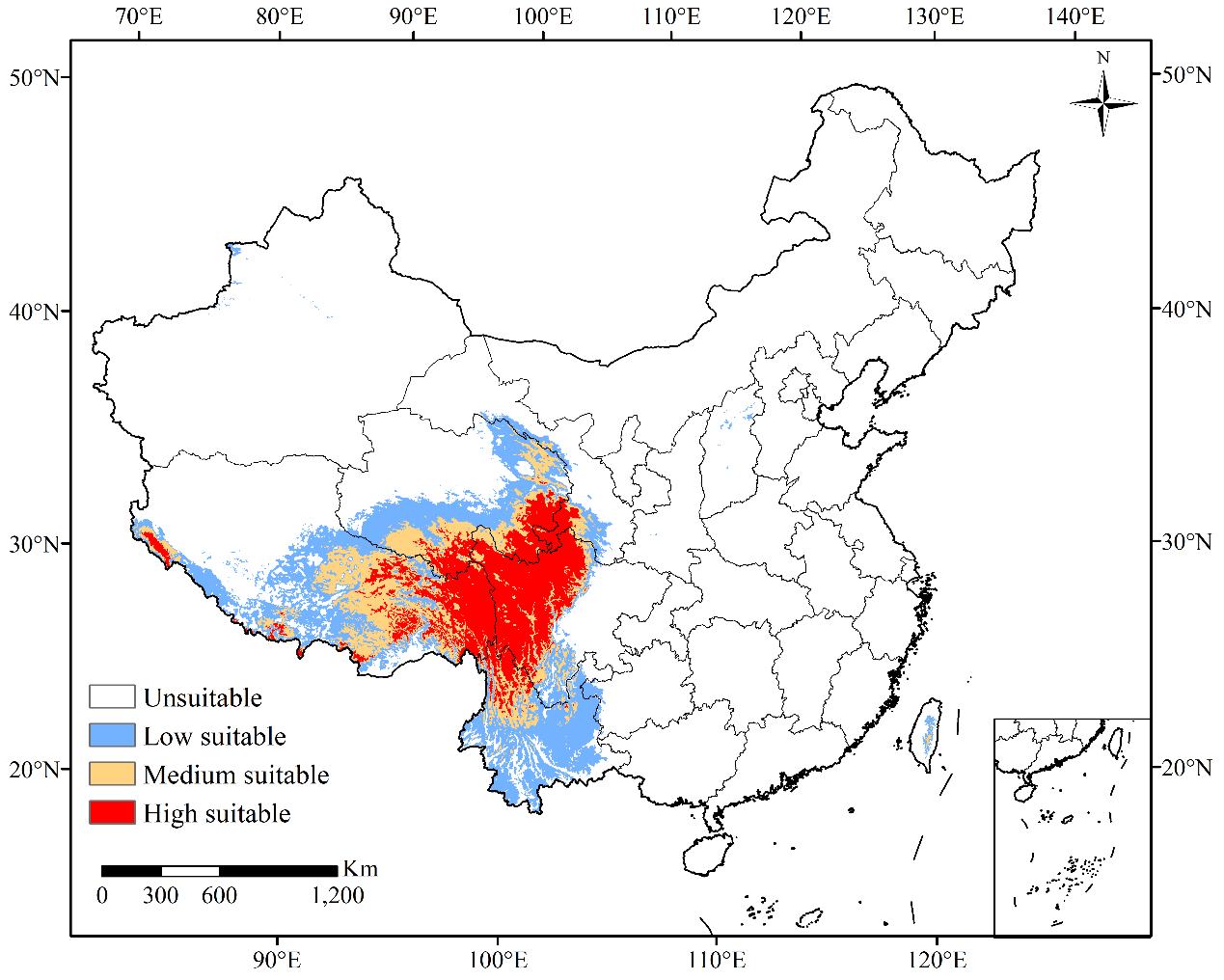

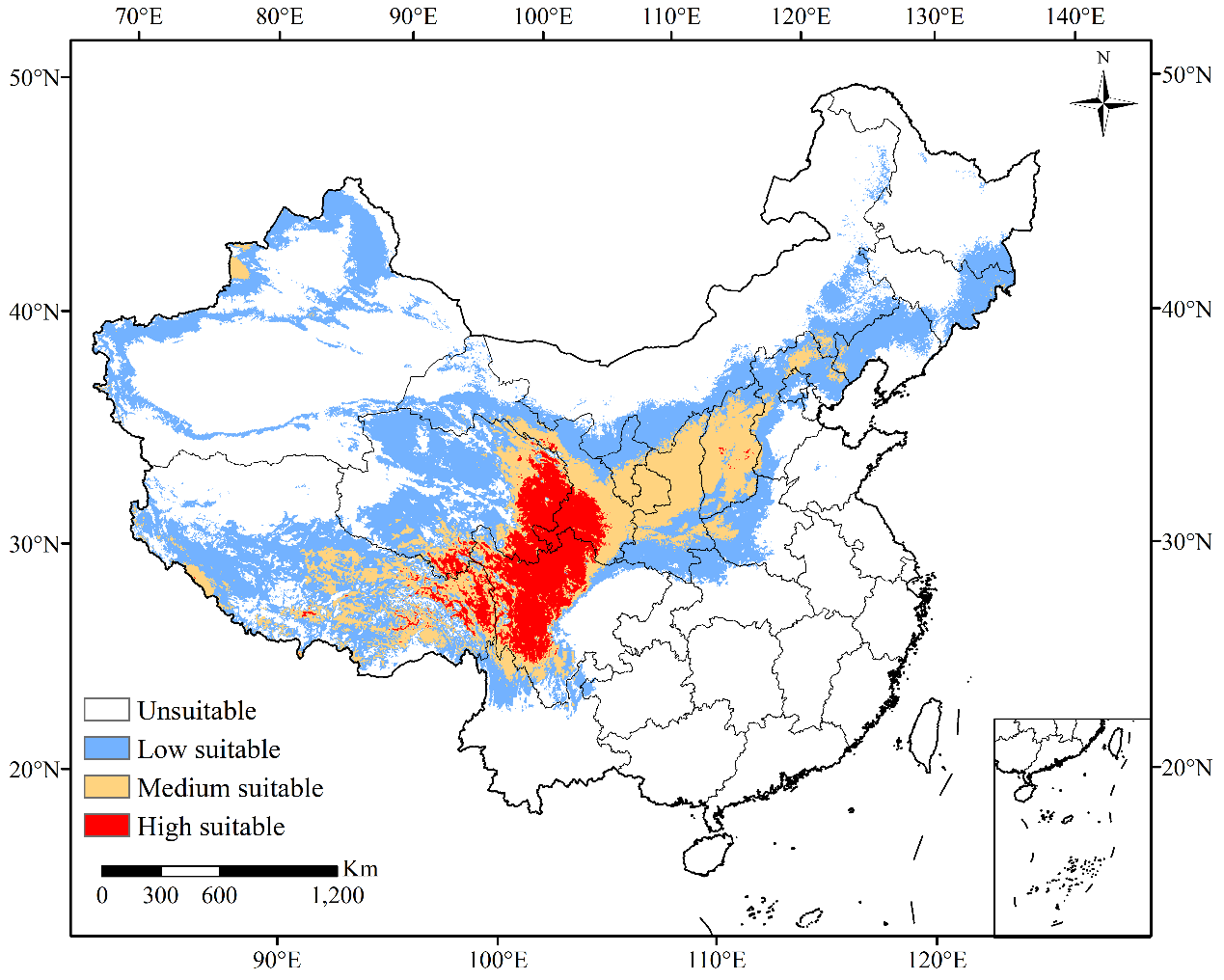

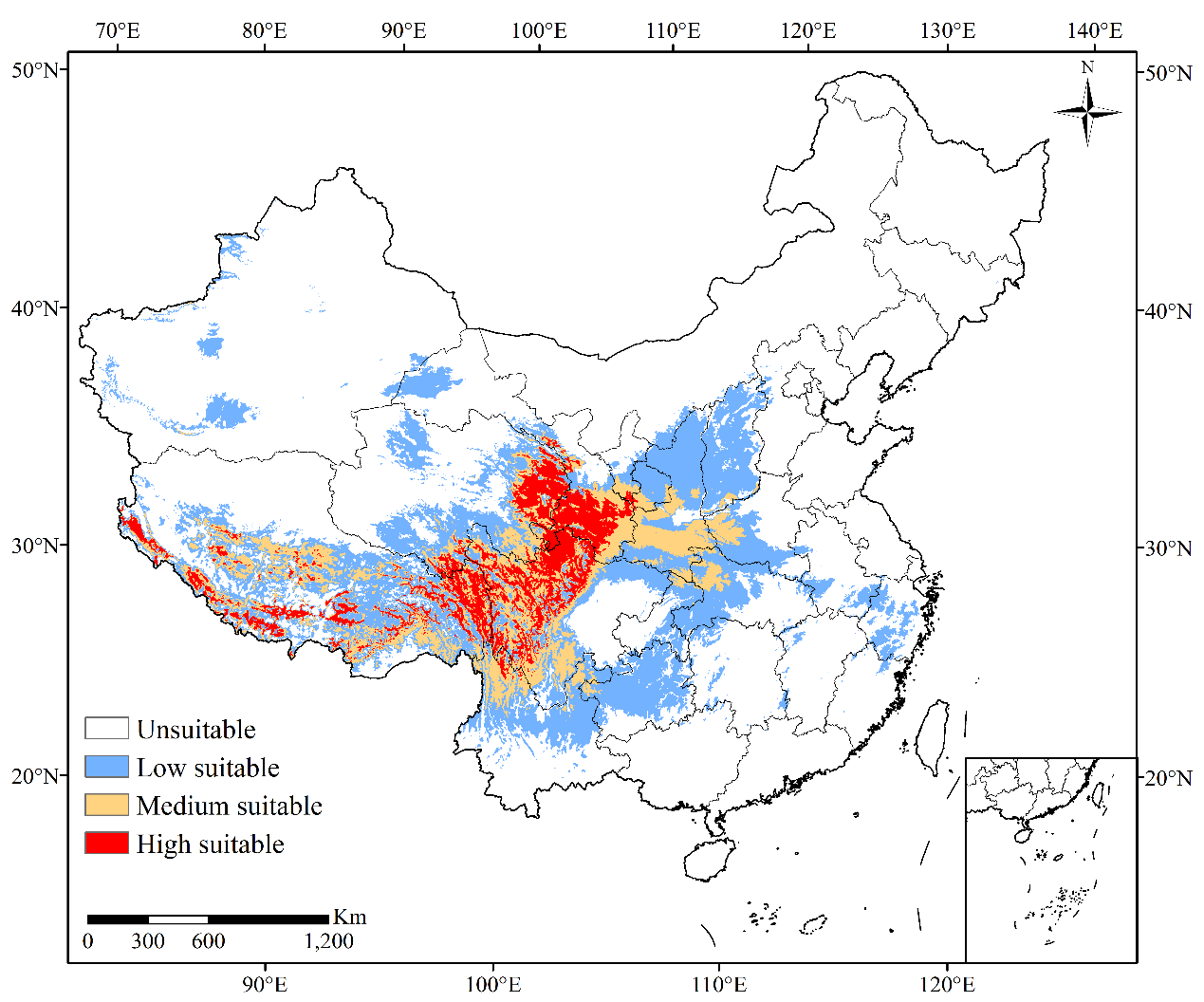


Fig.S3 Potential distribution of *Aster diplostephioides* in China based on Maxent under current climate.

Fig.S4 Potential distribution of *Aster farreri* in China based on Maxent under current climate.

Fig.S5 Potential distribution of *Aster poliothamnus* in China based on Maxent under current climate.


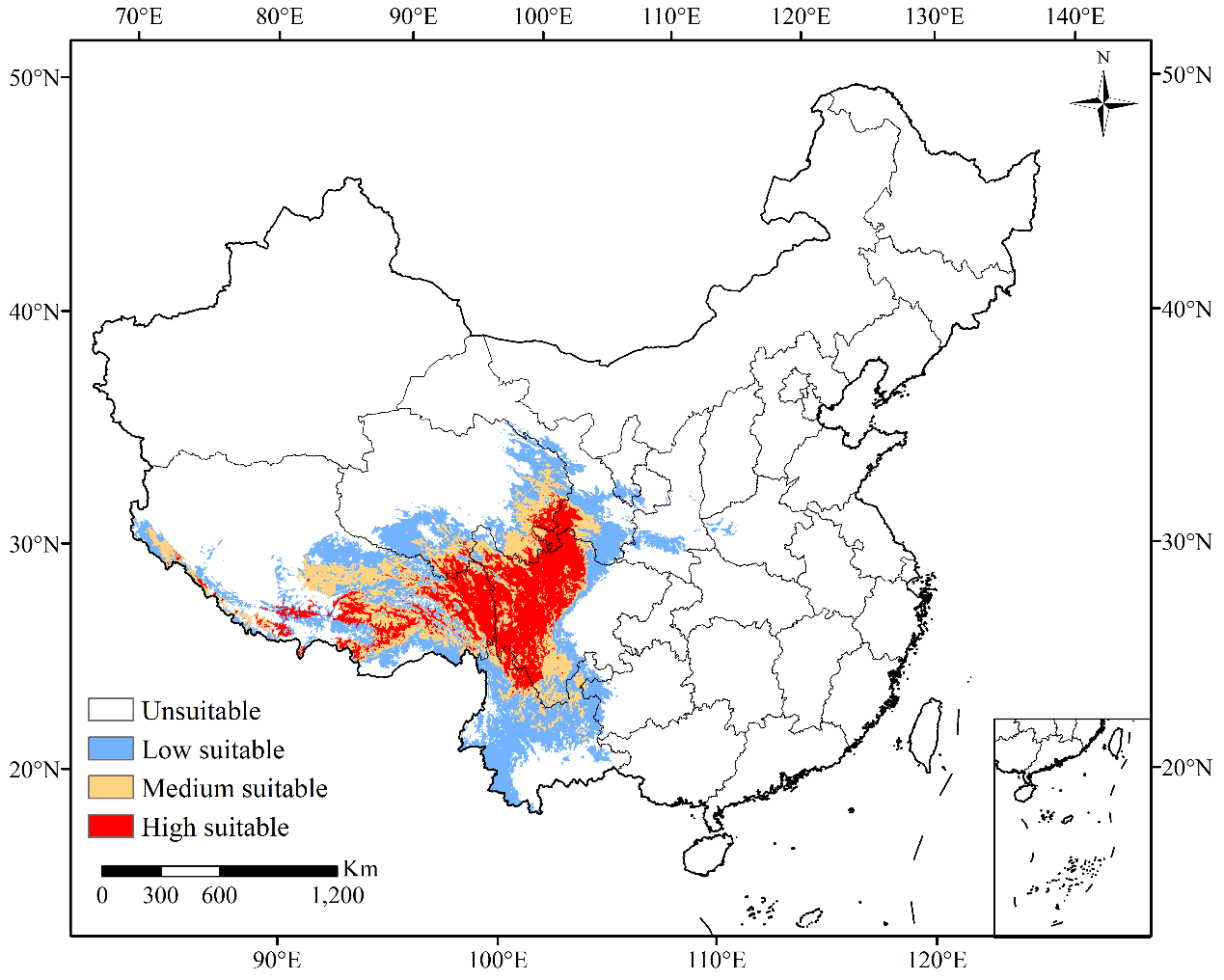


Fig.S6 Potential distribution of *Aster souliei* in China based on Maxent under current climate.


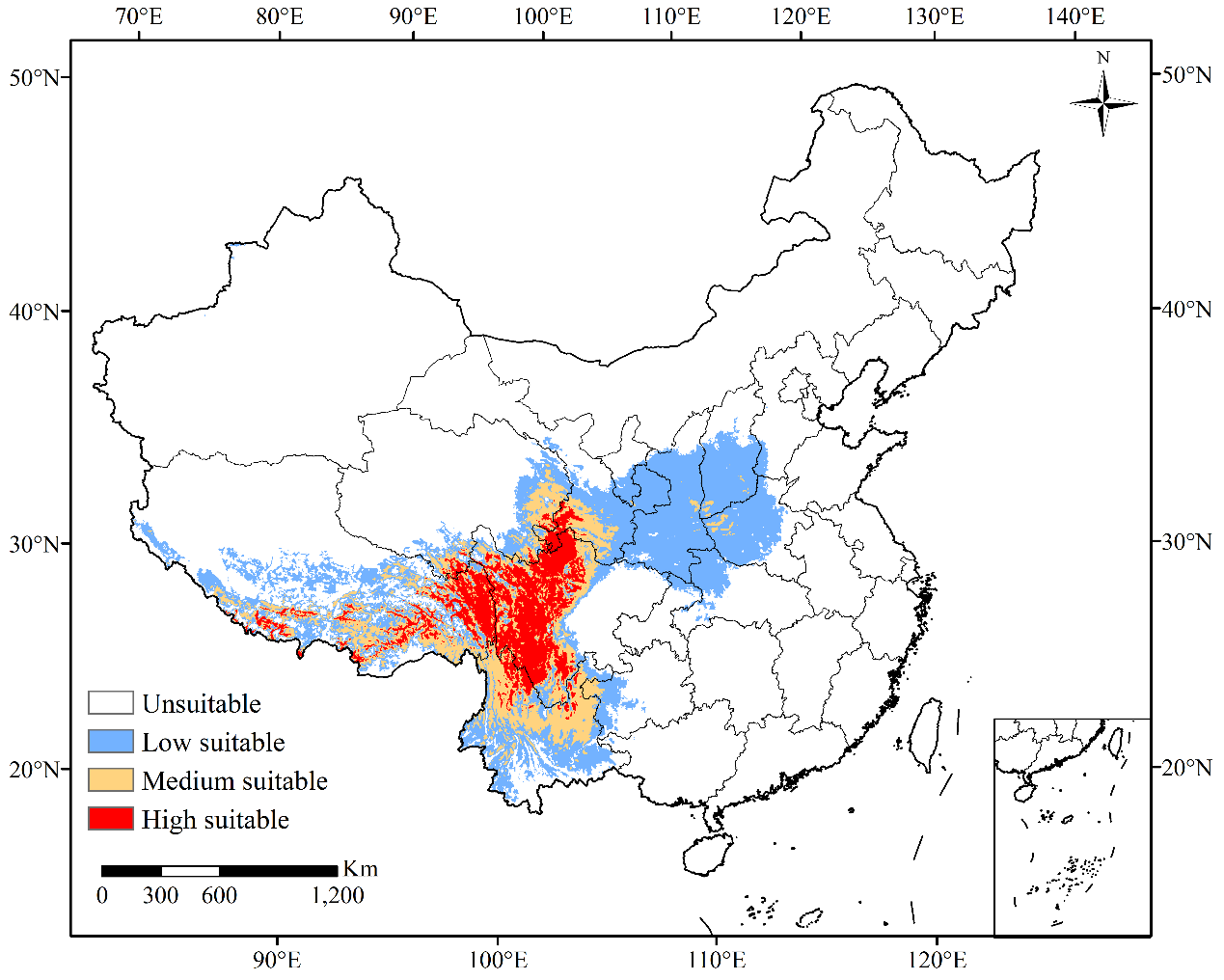


Fig.S7 Potential distribution of *Aster tongolensis* in China based on Maxent under current climate.


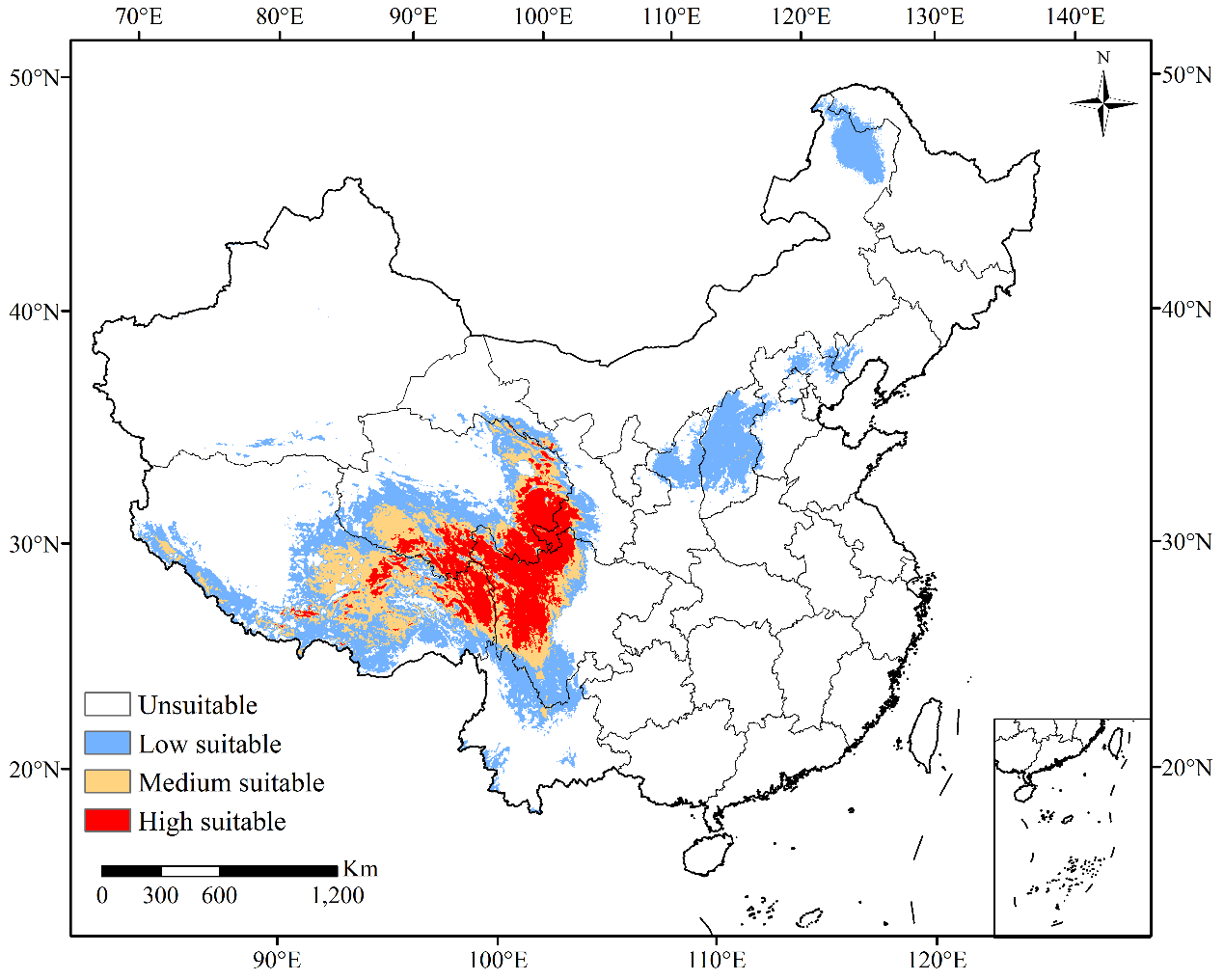

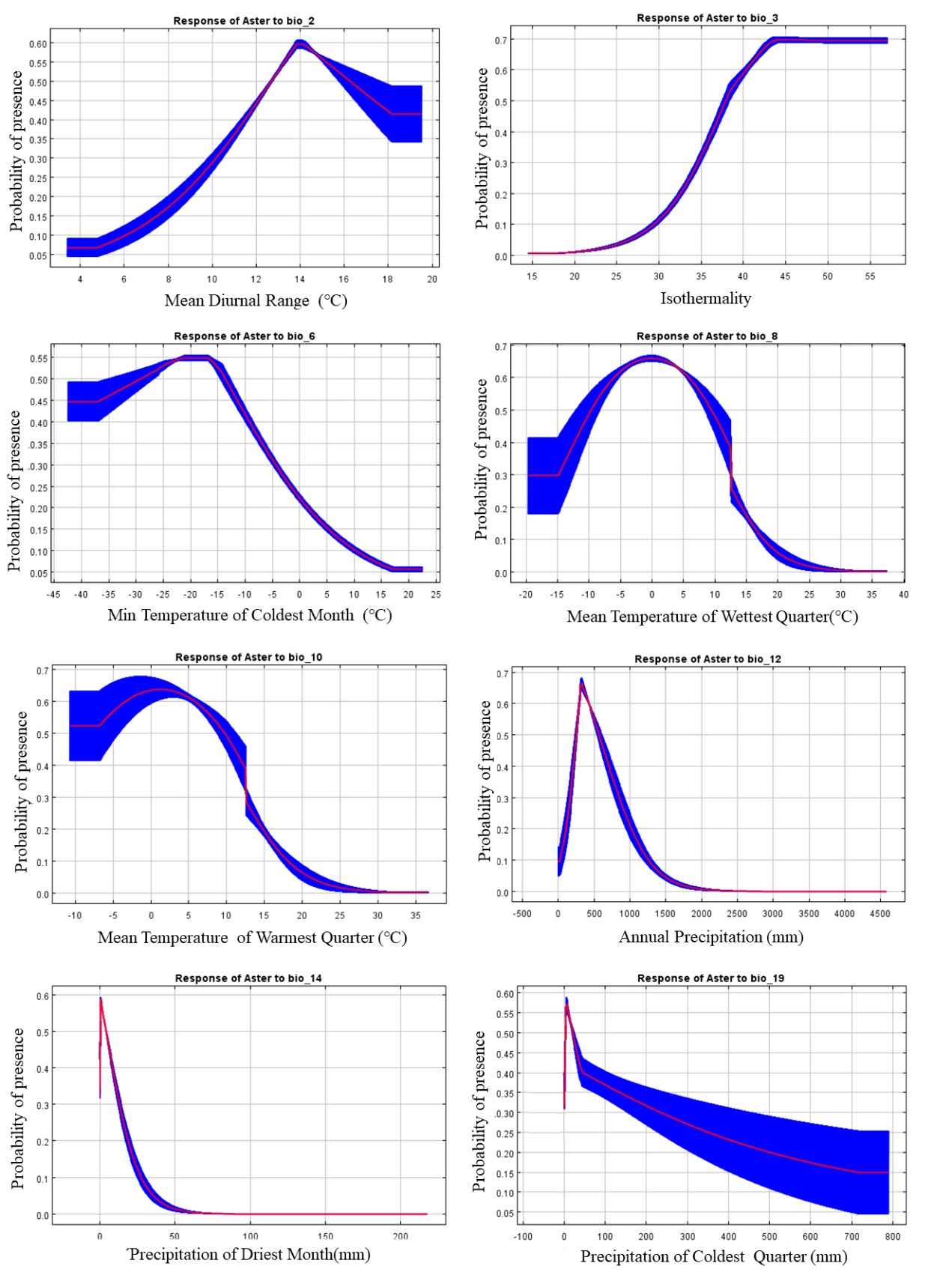


Fig.S8 Predictions of the potentially suitable area of *Aster yunnanensis* var*. labrangensis* under current climate conditions based on the MaxEnt model.

Fig.S9 The response curves on dominant environmental variables of *Asterasteroides.*


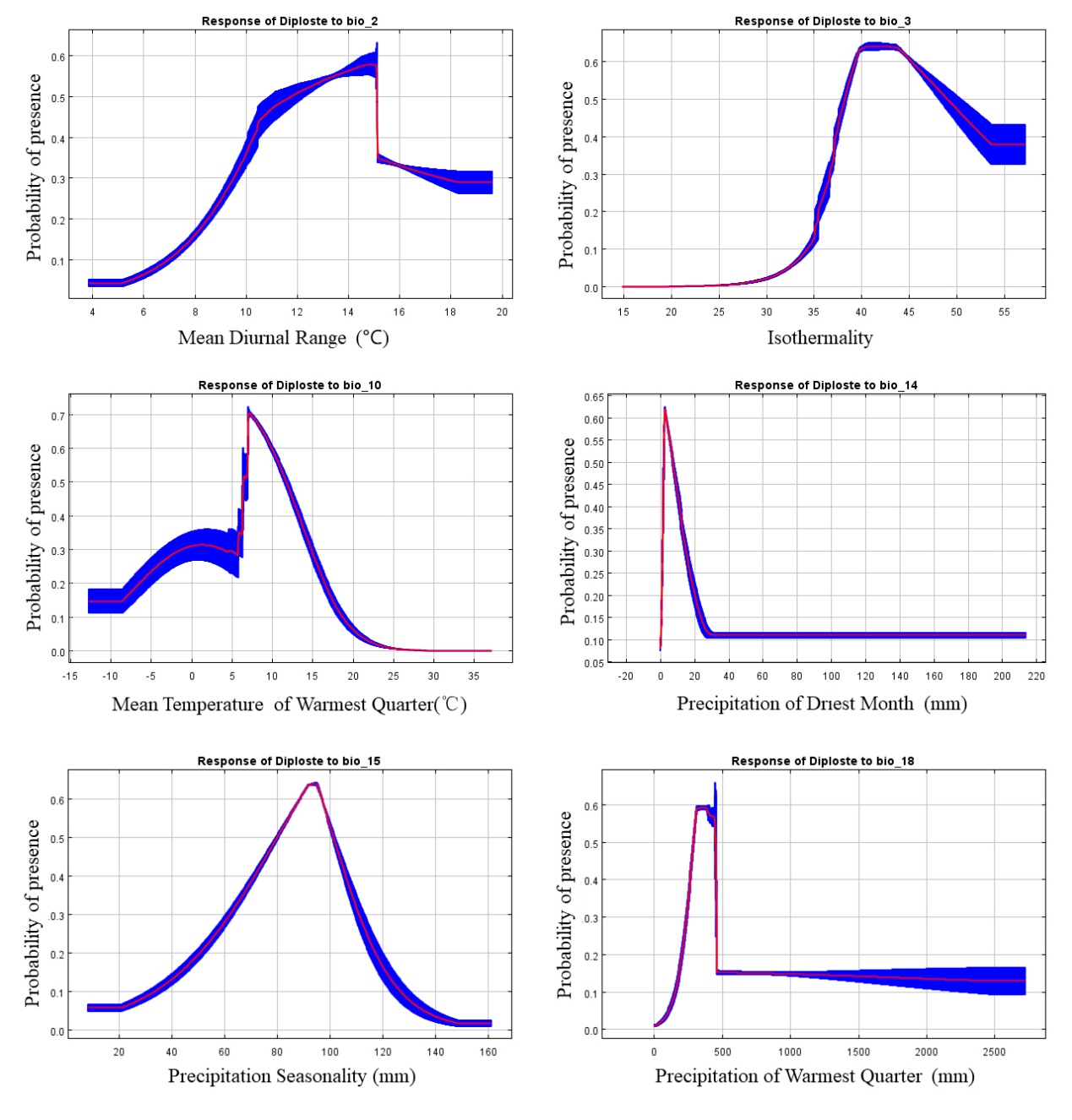


Fig.S10 The response curves on dominant environmental variables of *Aster diplostephioides.*


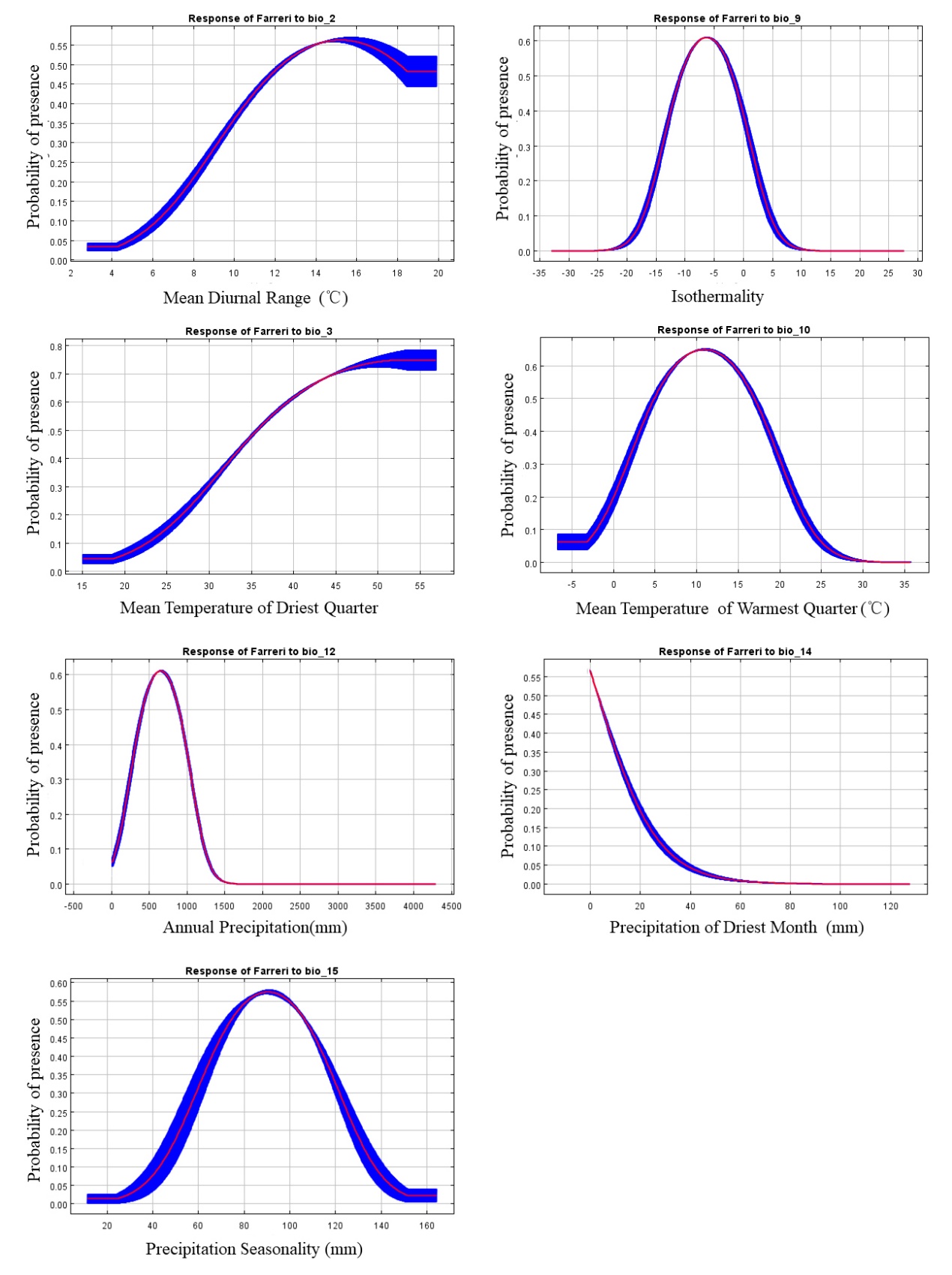

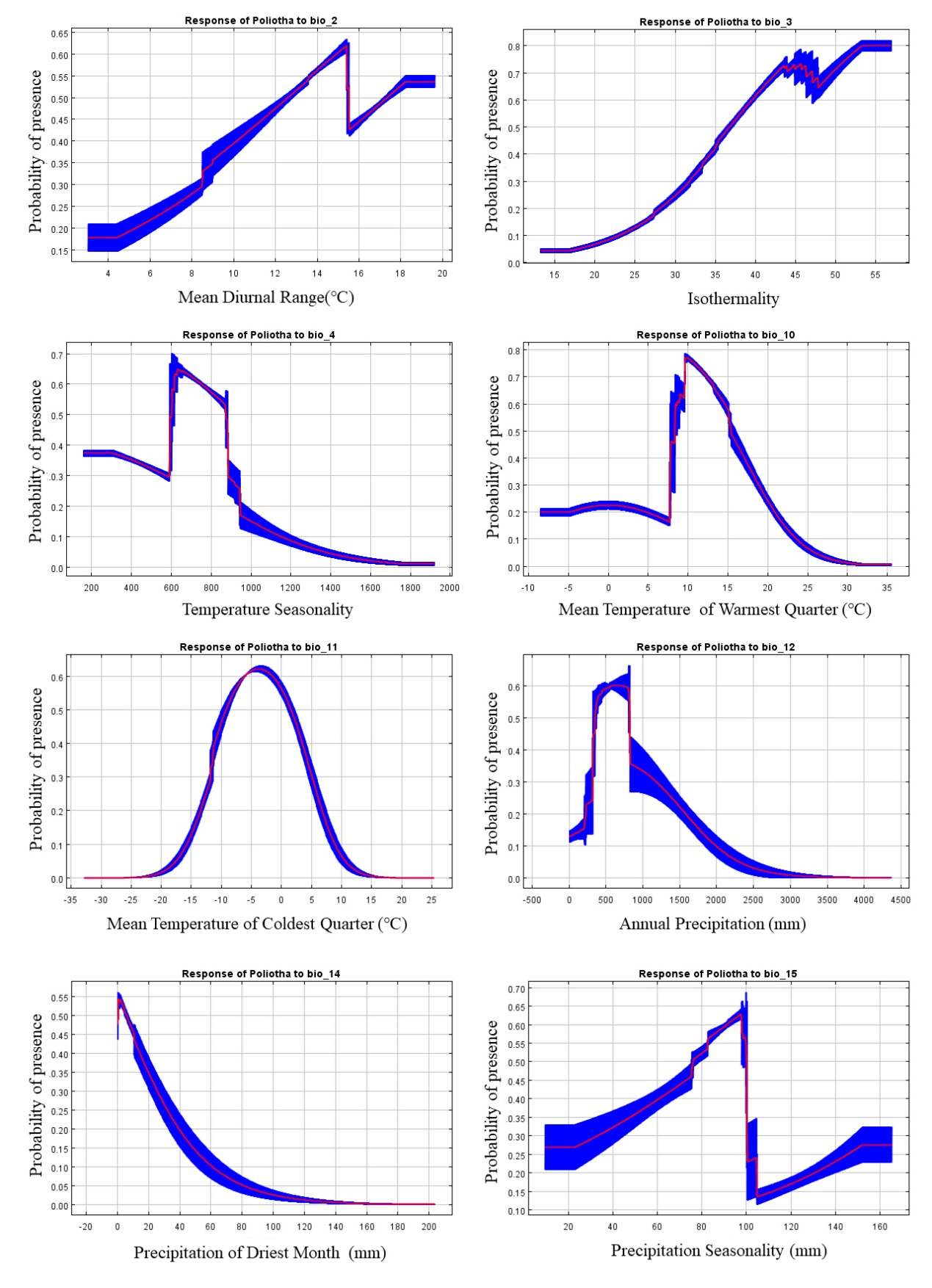


Fig.S11 The response curves on dominant environmental variables of *Aster farreri.*

Fig.S12 The response curves on dominant environmental variables of *Aster poliothamnus*.


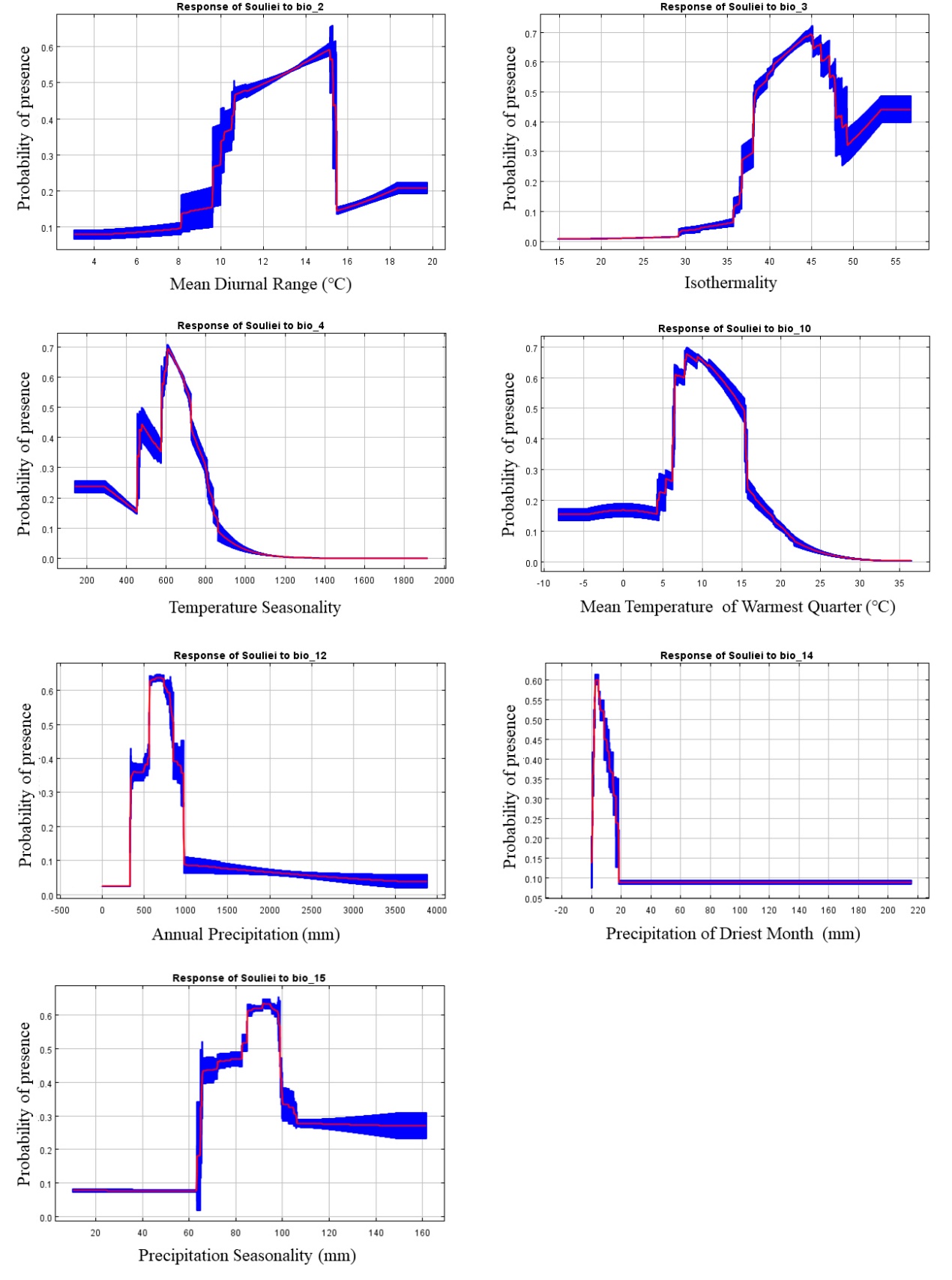


Fig.S13 The response curves on dominant environmental variables of *Aster souliei.*


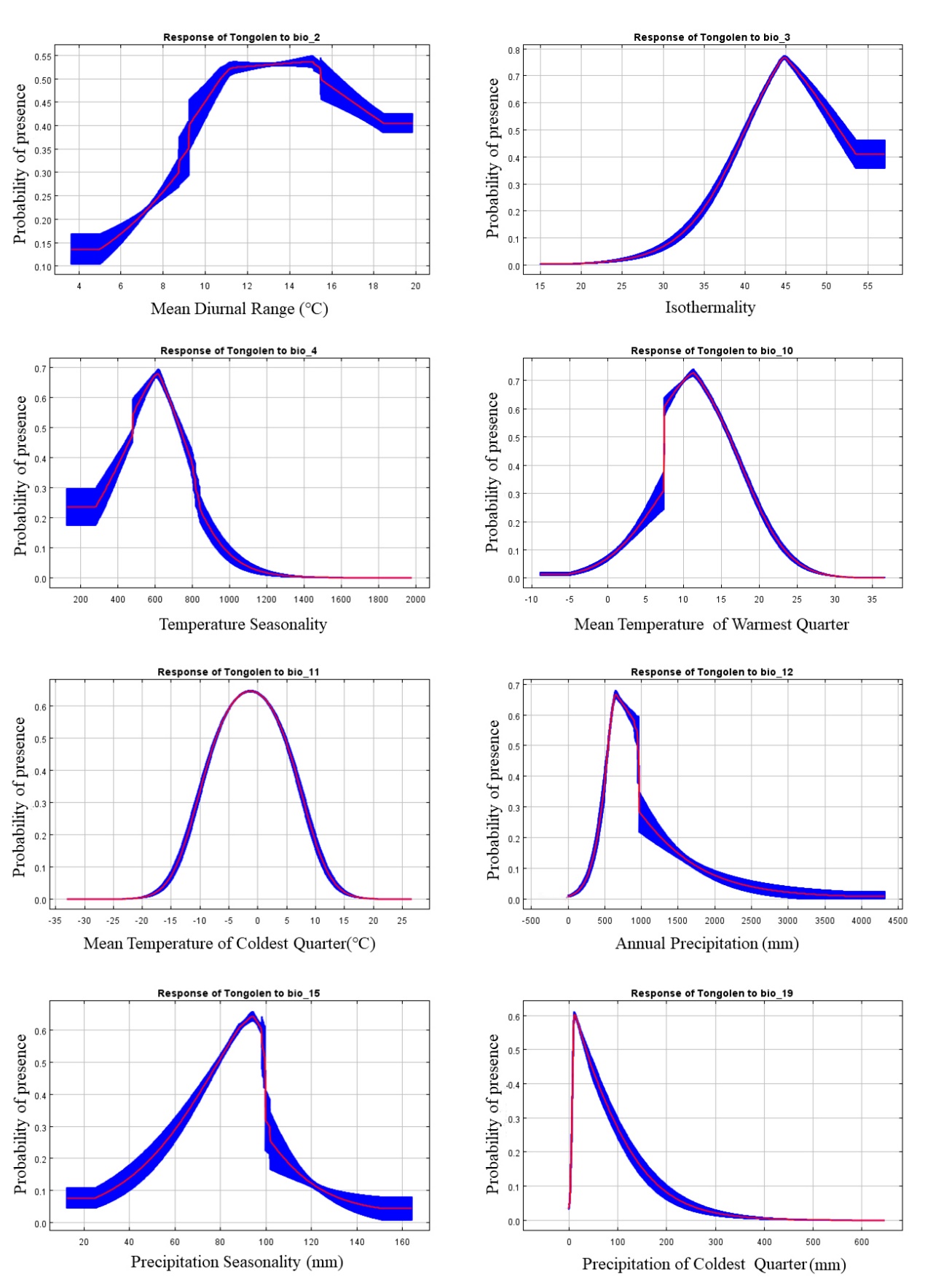

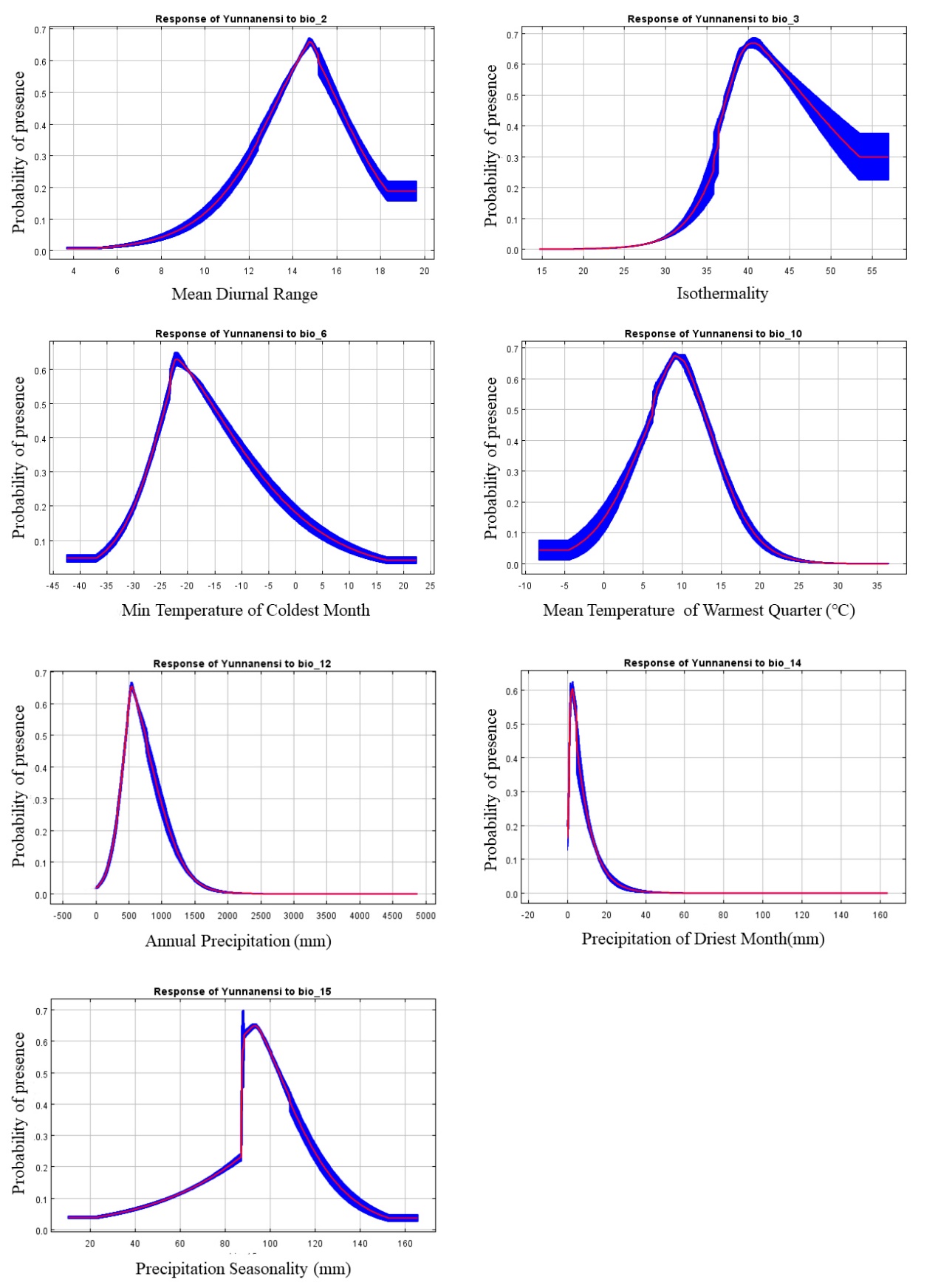

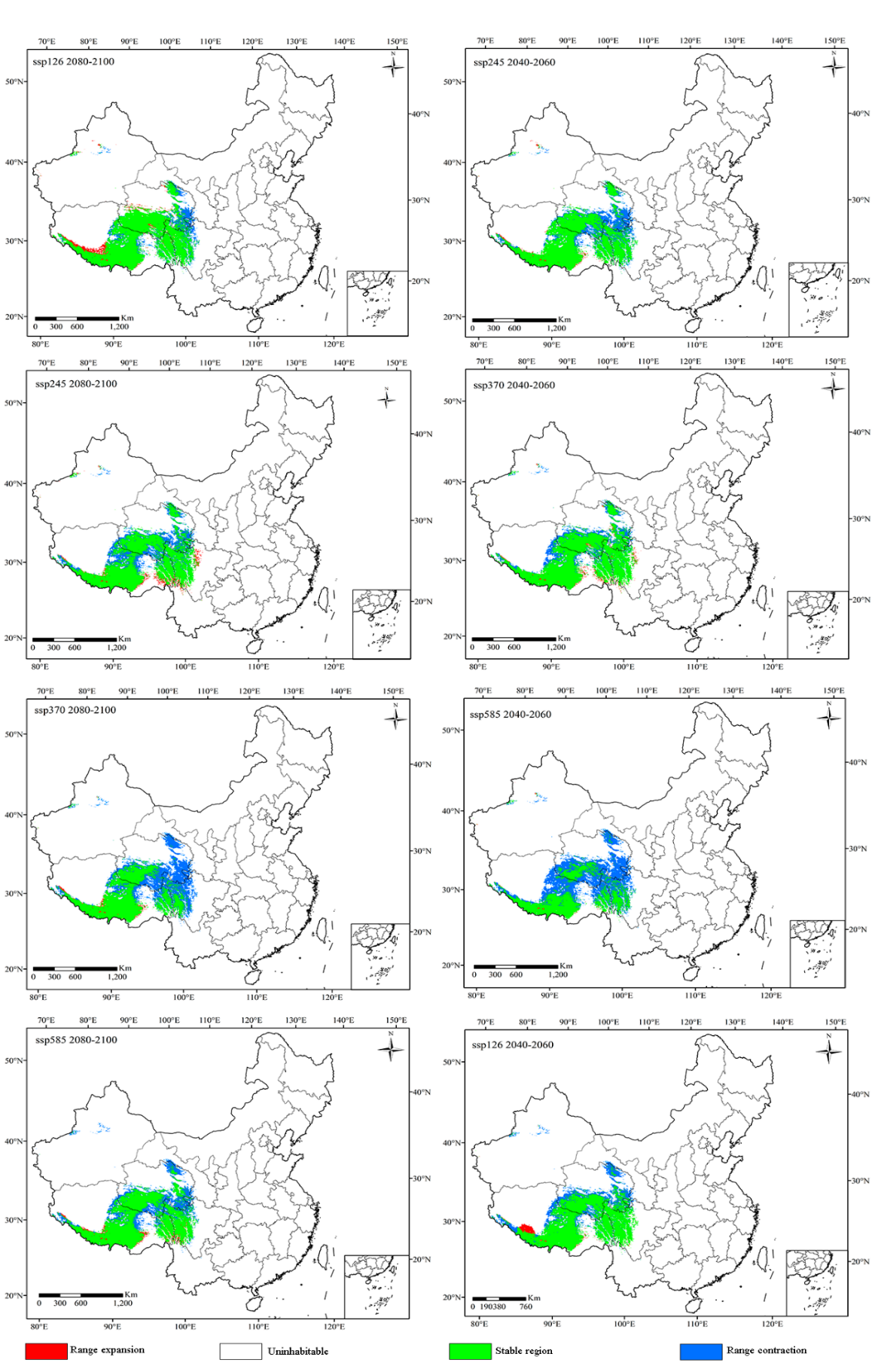


Fig.S14 The response curves on dominant environmental variables of *Aster tongolensis*.

Fig.S15 The response curves on dominant environmental variables of *Aster yunnanensis* var*. labrangensis.*

Fig.S16 Changes of potential geographical distribution of *Aster asteroides* under climate change scenarios in the future

Note: Red: Range expansion; White: Unhabitable; Green: Stable region; Blue: Range contraction. The same below


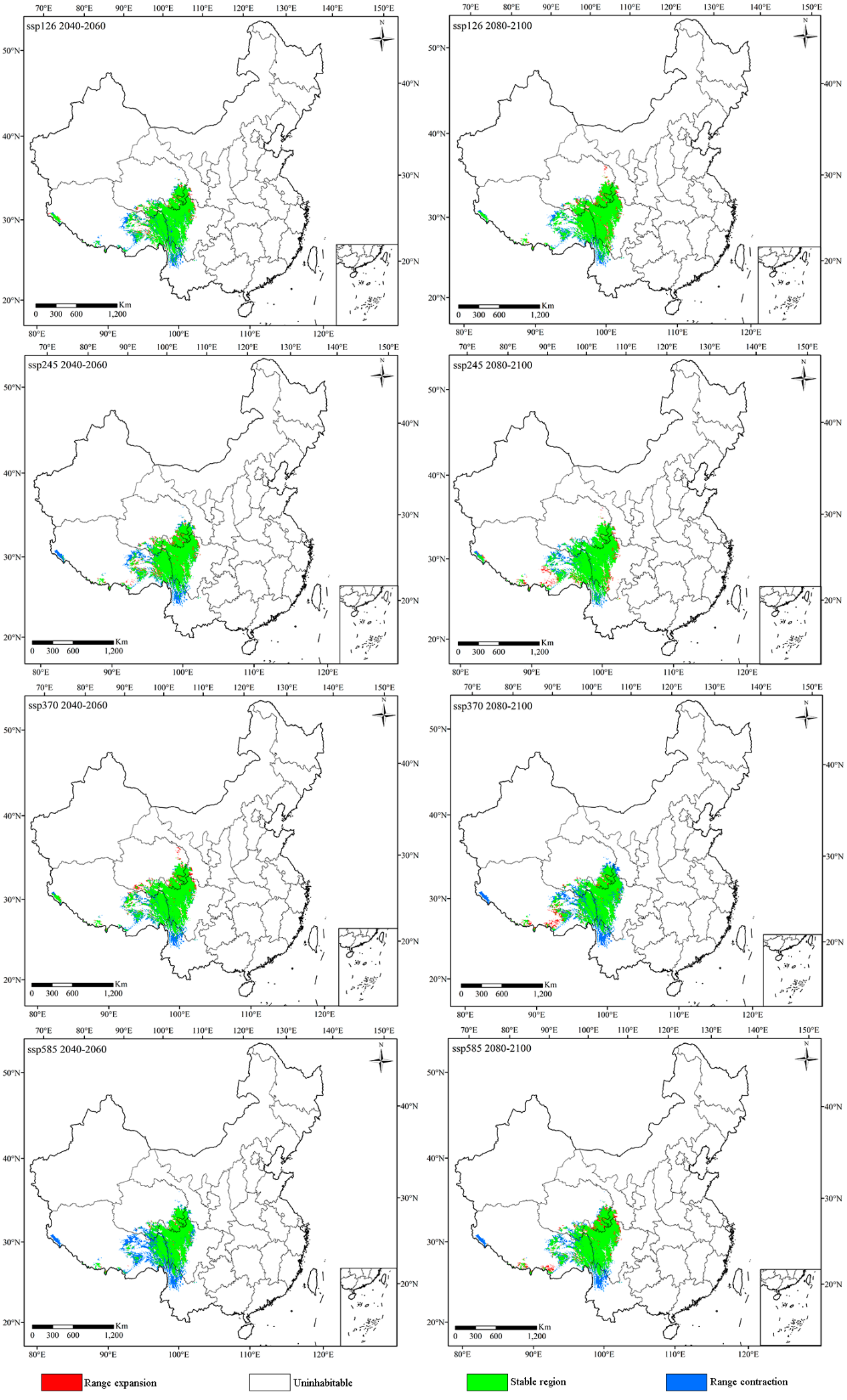


Fig.S17 Changes of potential geographical distribution of *Aster diplostephioides* under climate change scenarios in the future


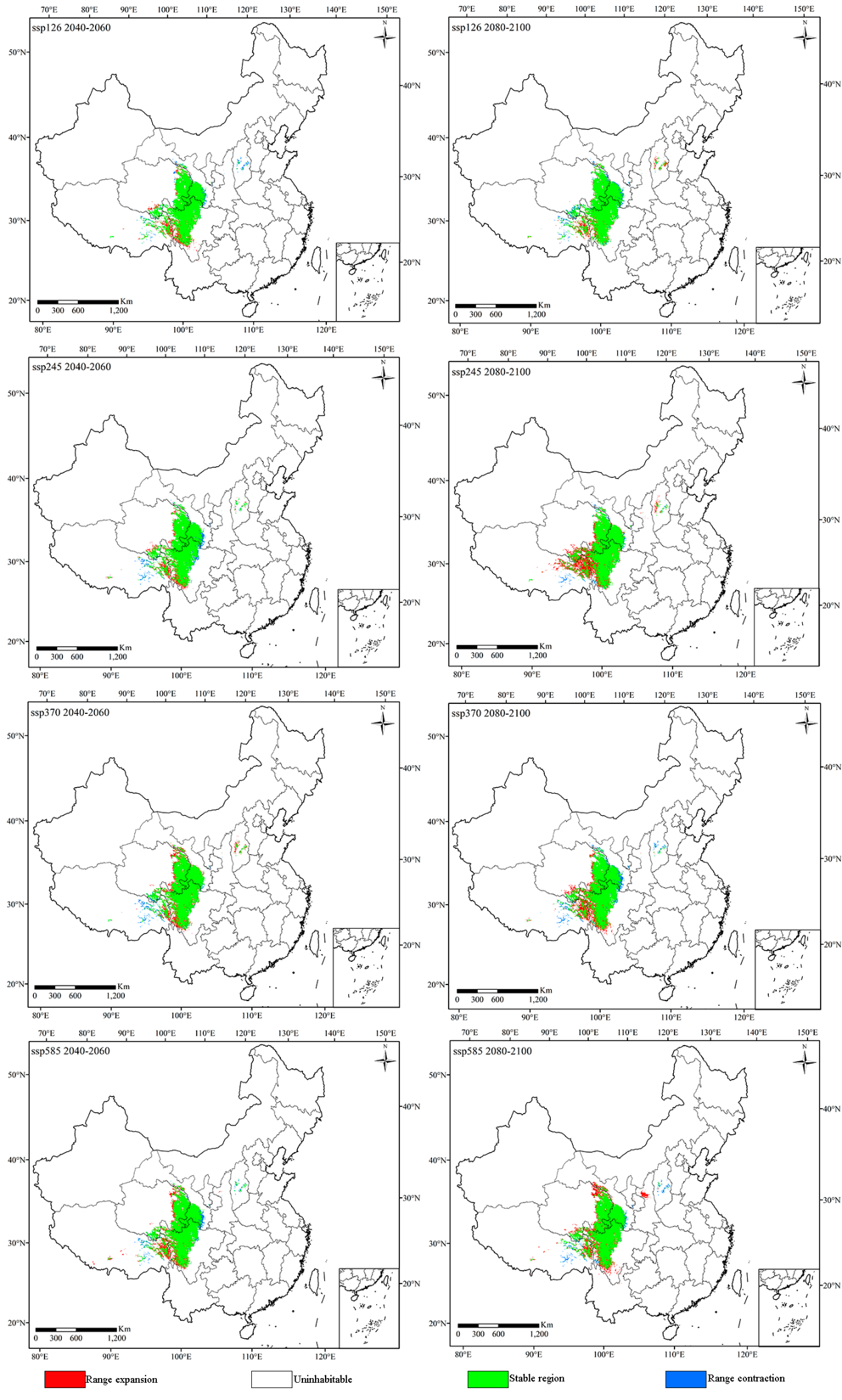


Fig.S18 Changes of potential geographical distribution of *Aster farreri* under climate change scenarios in the future


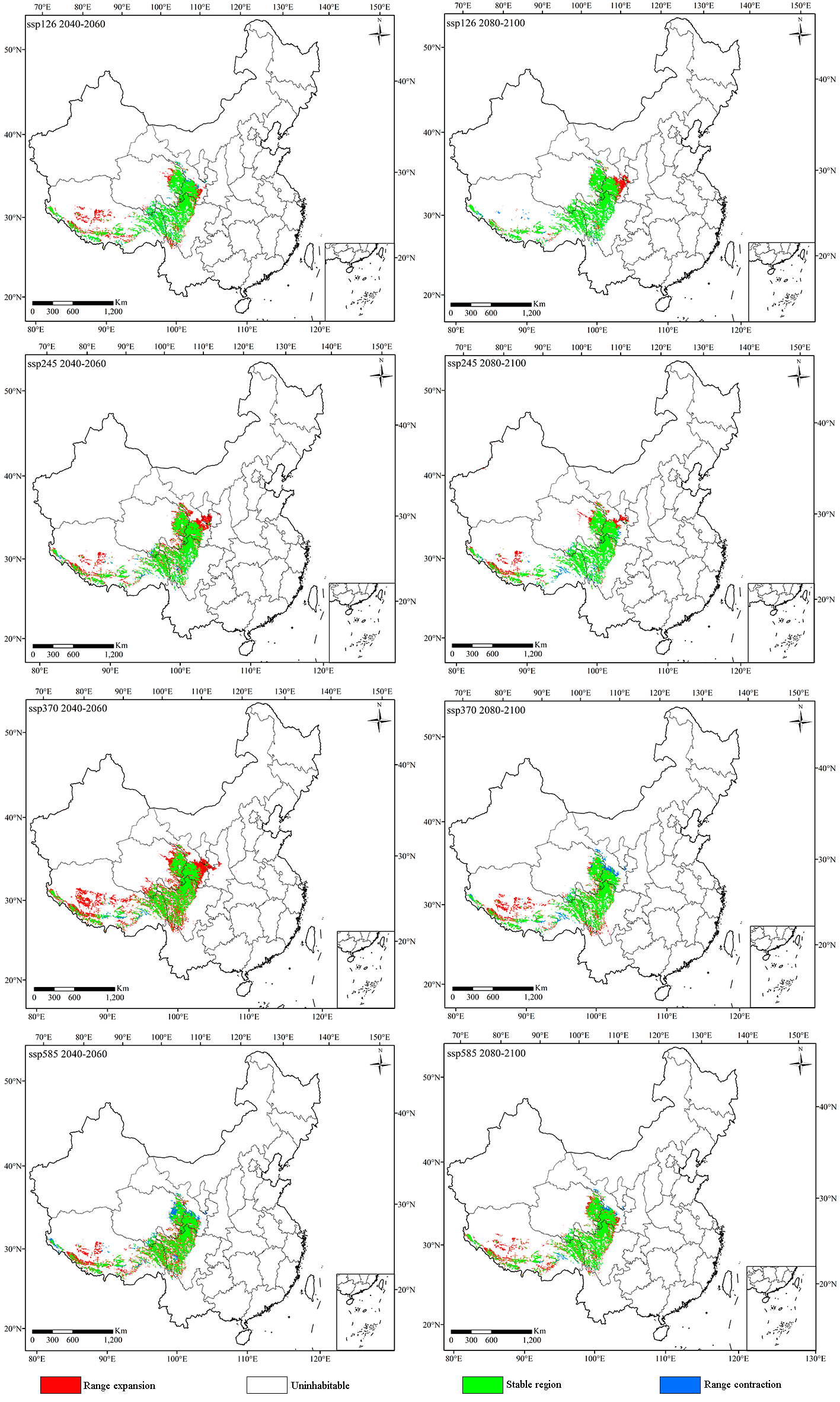
Fig.S19 Changes of potential geographical distribution of *Aster poliothamnus* under climate change scenarios in the future


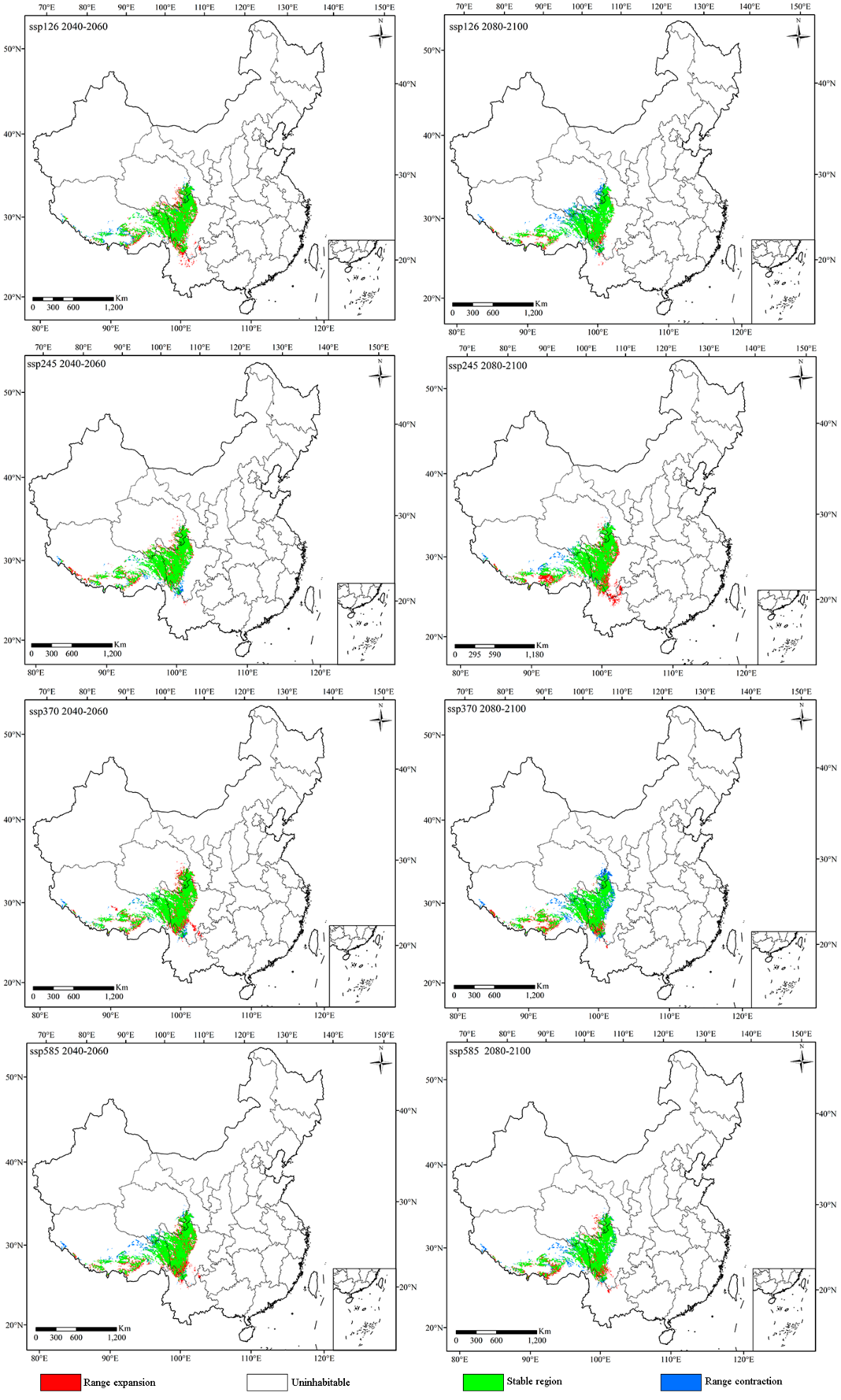


Fig.S20 Changes of potential geographical distribution of *Aster souliei* under climate change scenarios in the future


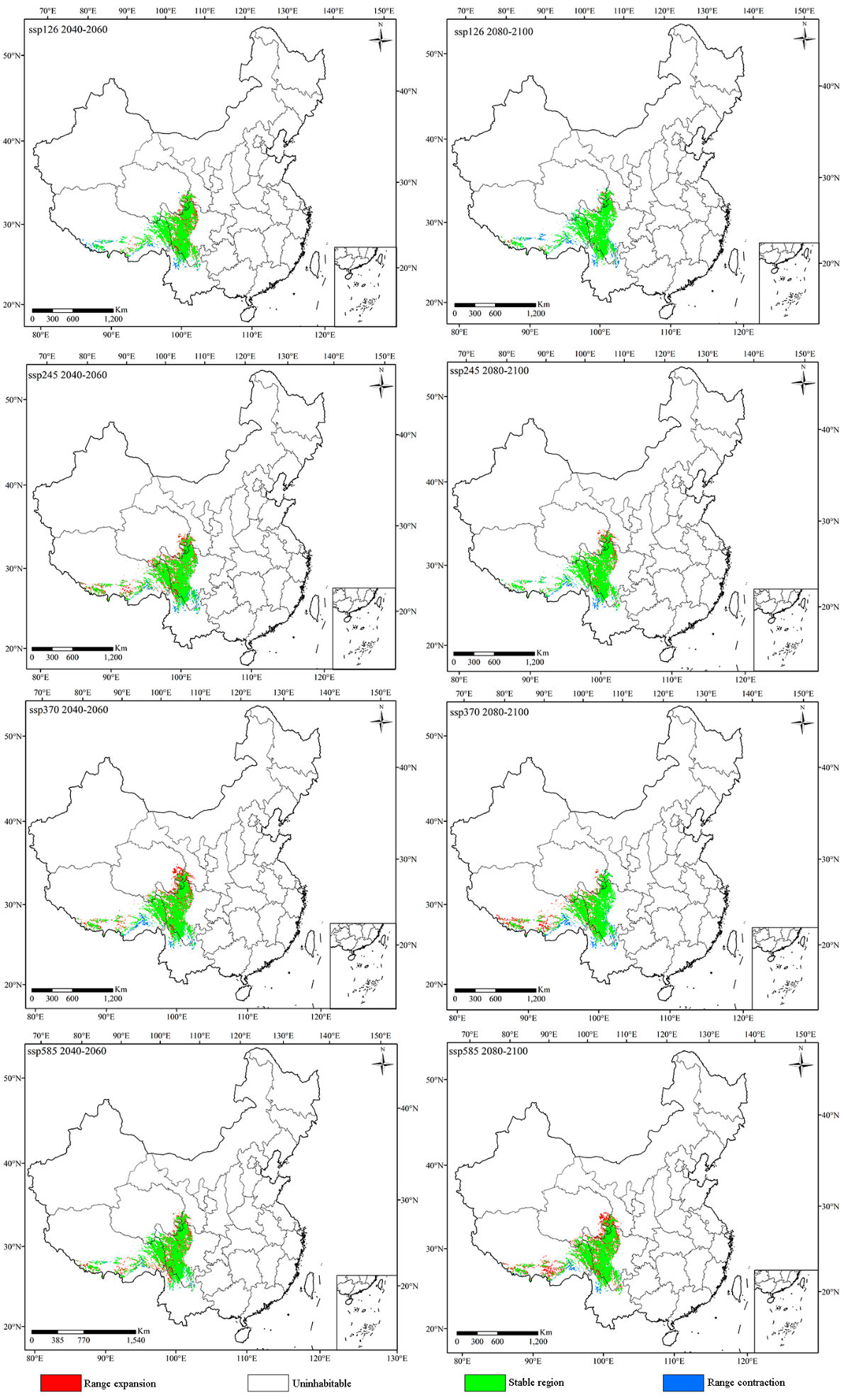


Fig.S21 Changes of potential geographical distribution of *Aster tongolensis* under climate change scenarios in the future


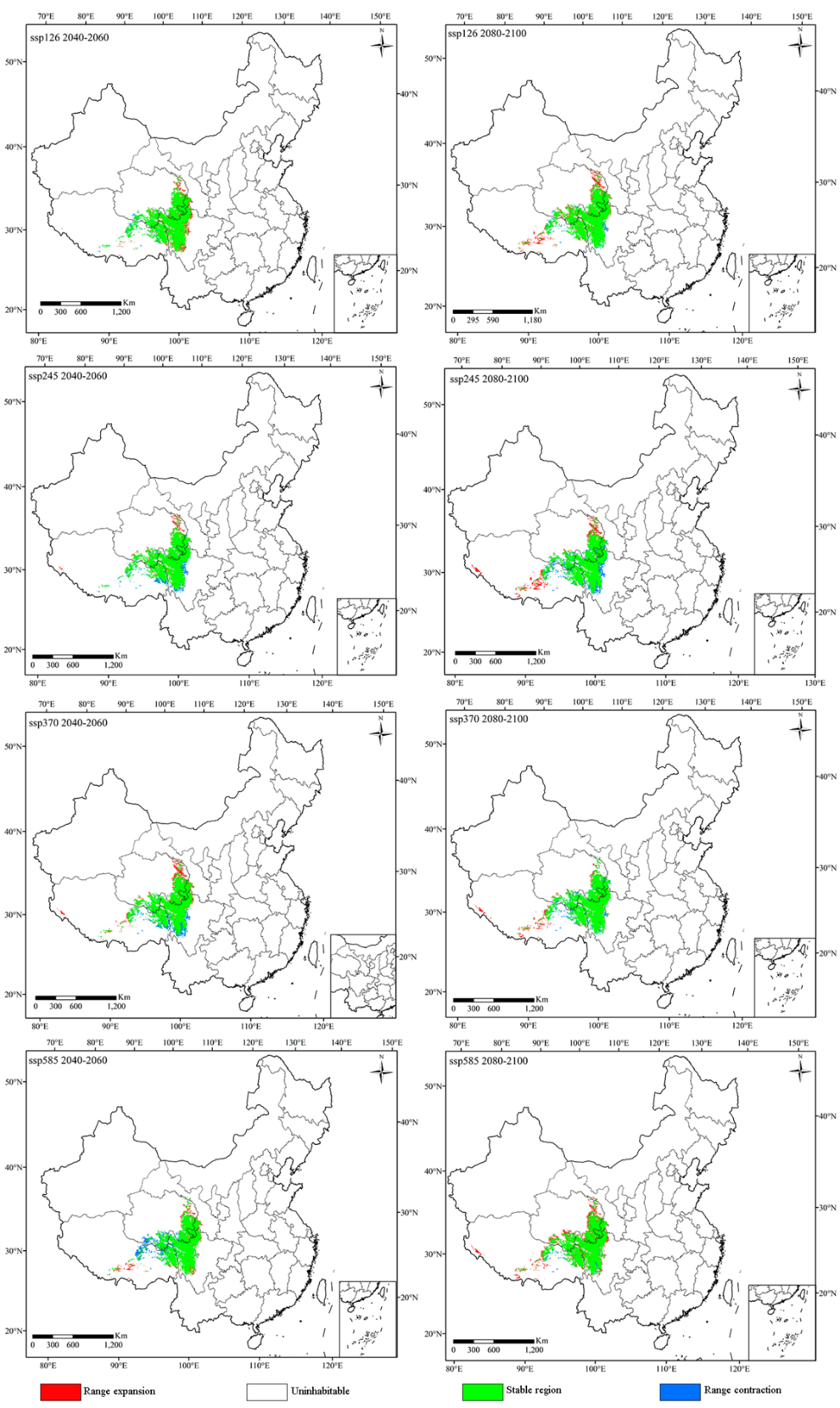

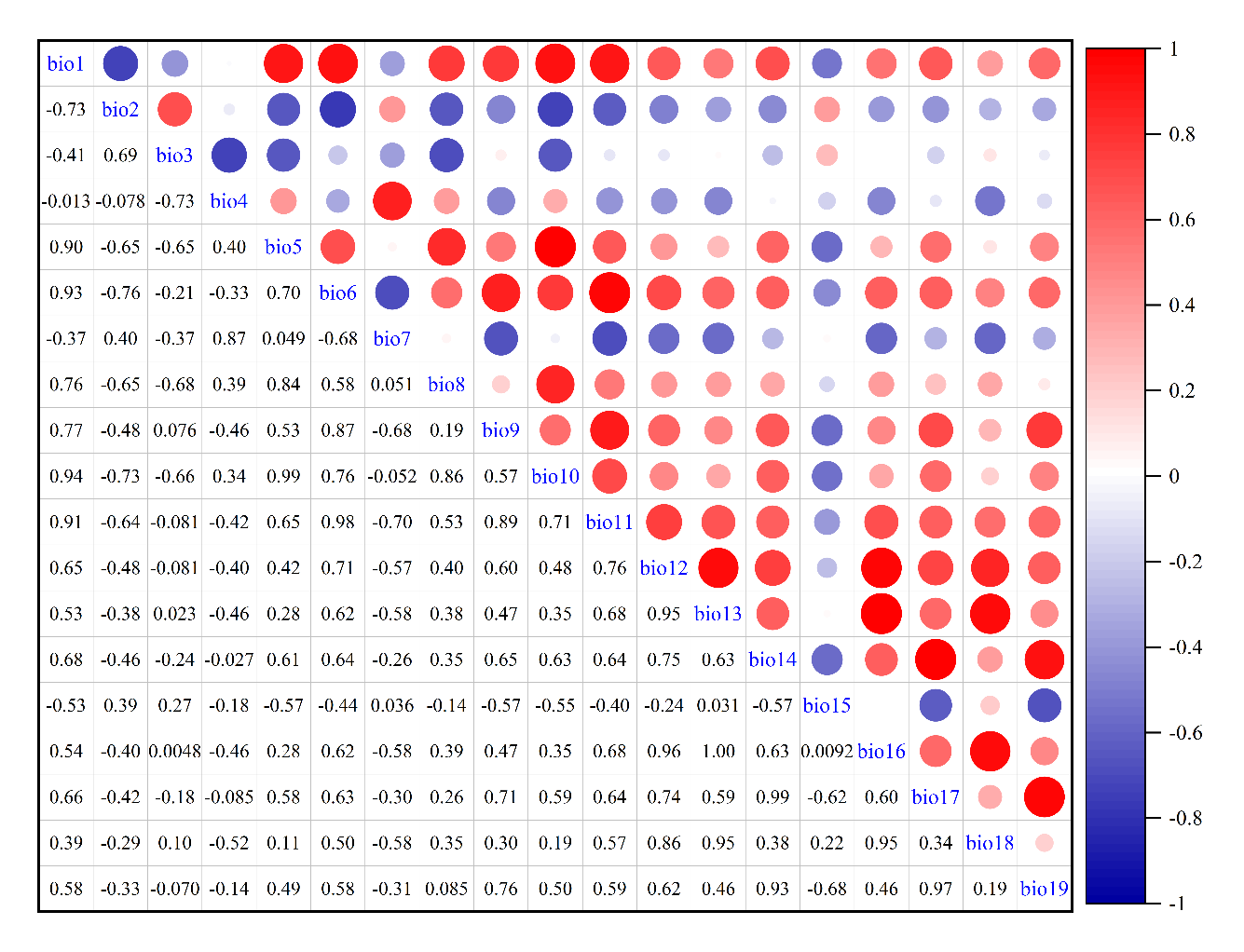


Fig.S22 Changes of potential geographical distribution of *Aster yunnanensis* var. *labrangensis* under climate change scenarios in the future.

Fig.S23 Correlation among 19 climate variables (*Aster asteroides*)


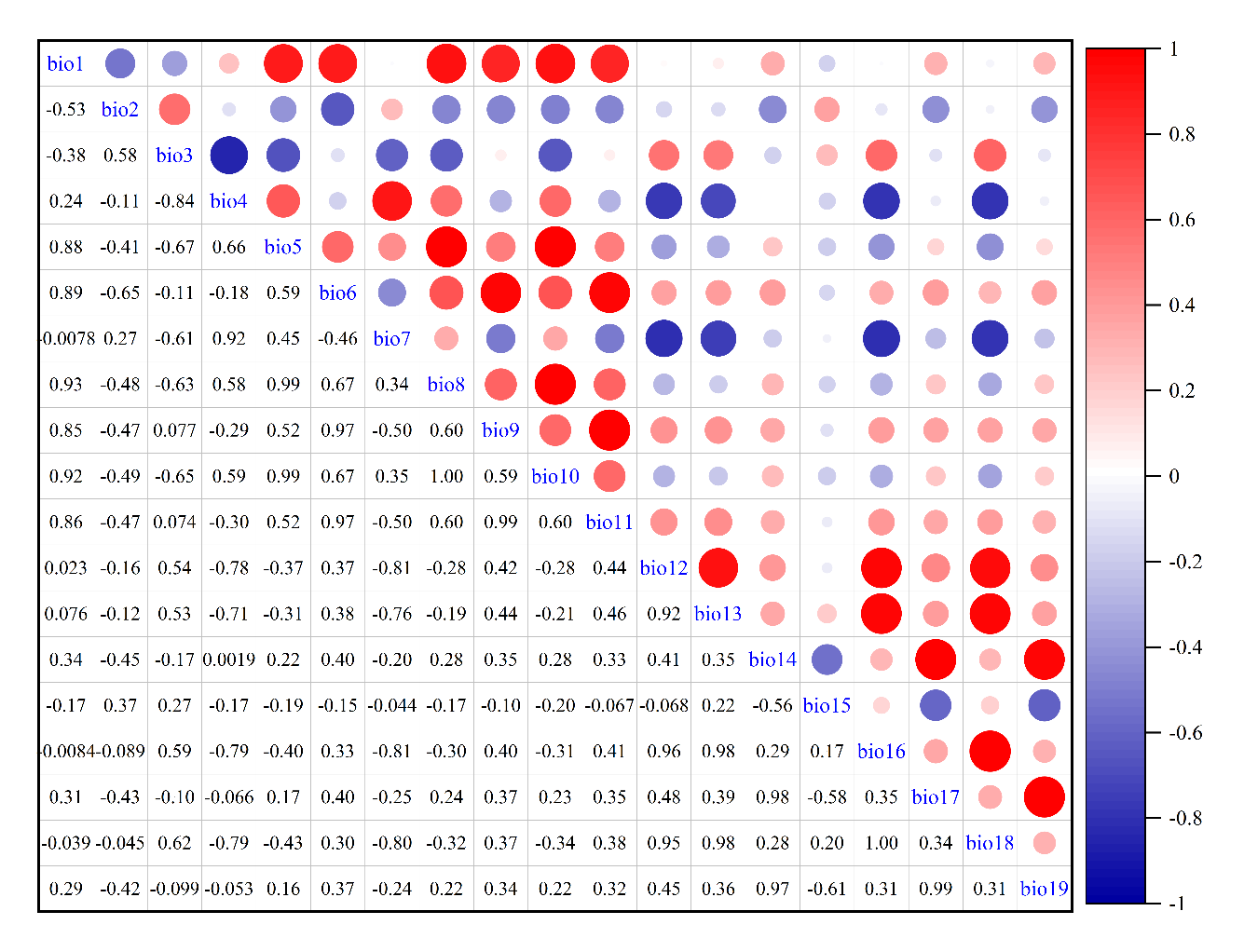


Fig.S24 Correlation among 19 climate variables (*Aster diplostephioides*)


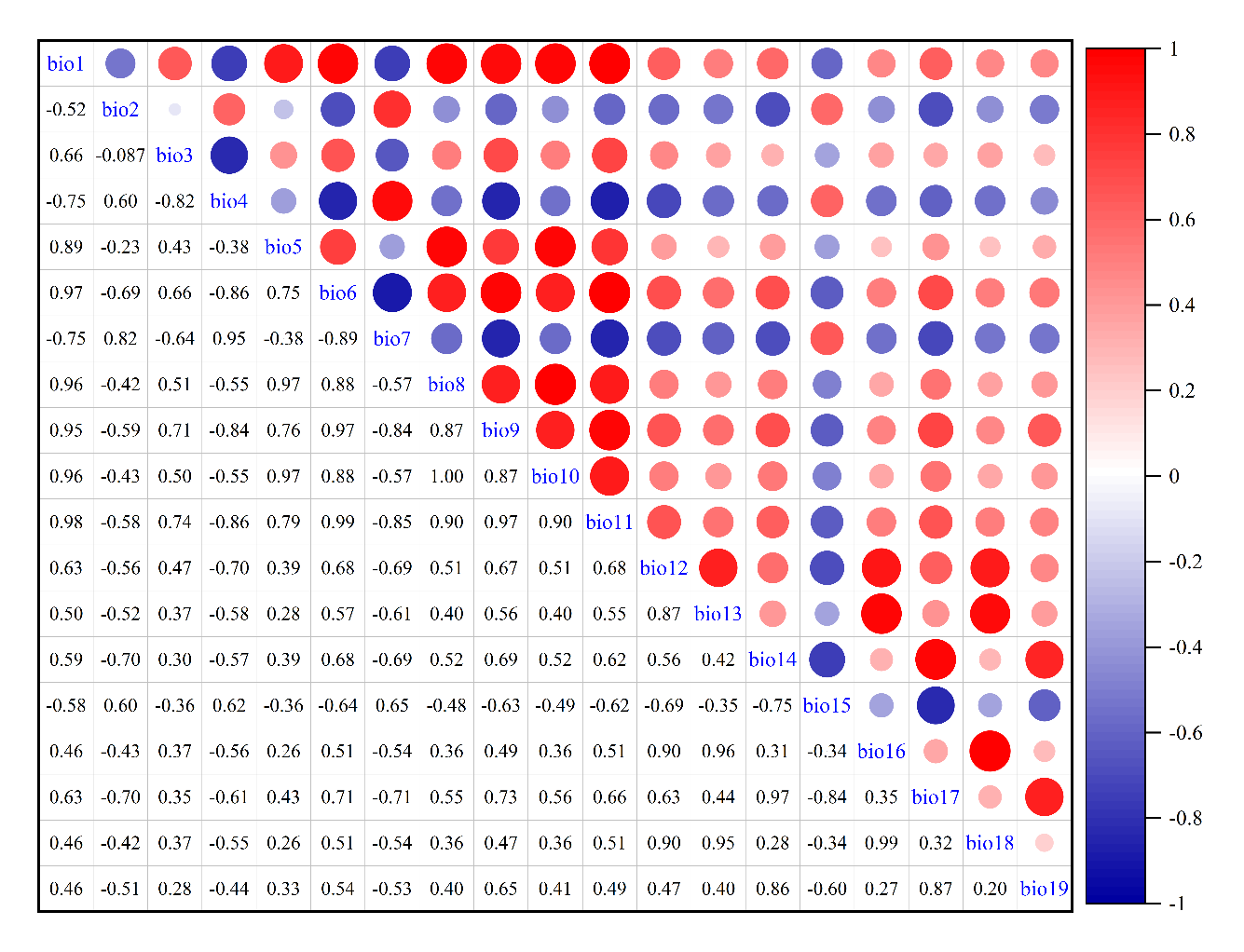


Fig.S25 Correlation among 19 climate variables (*Aster farreri*)


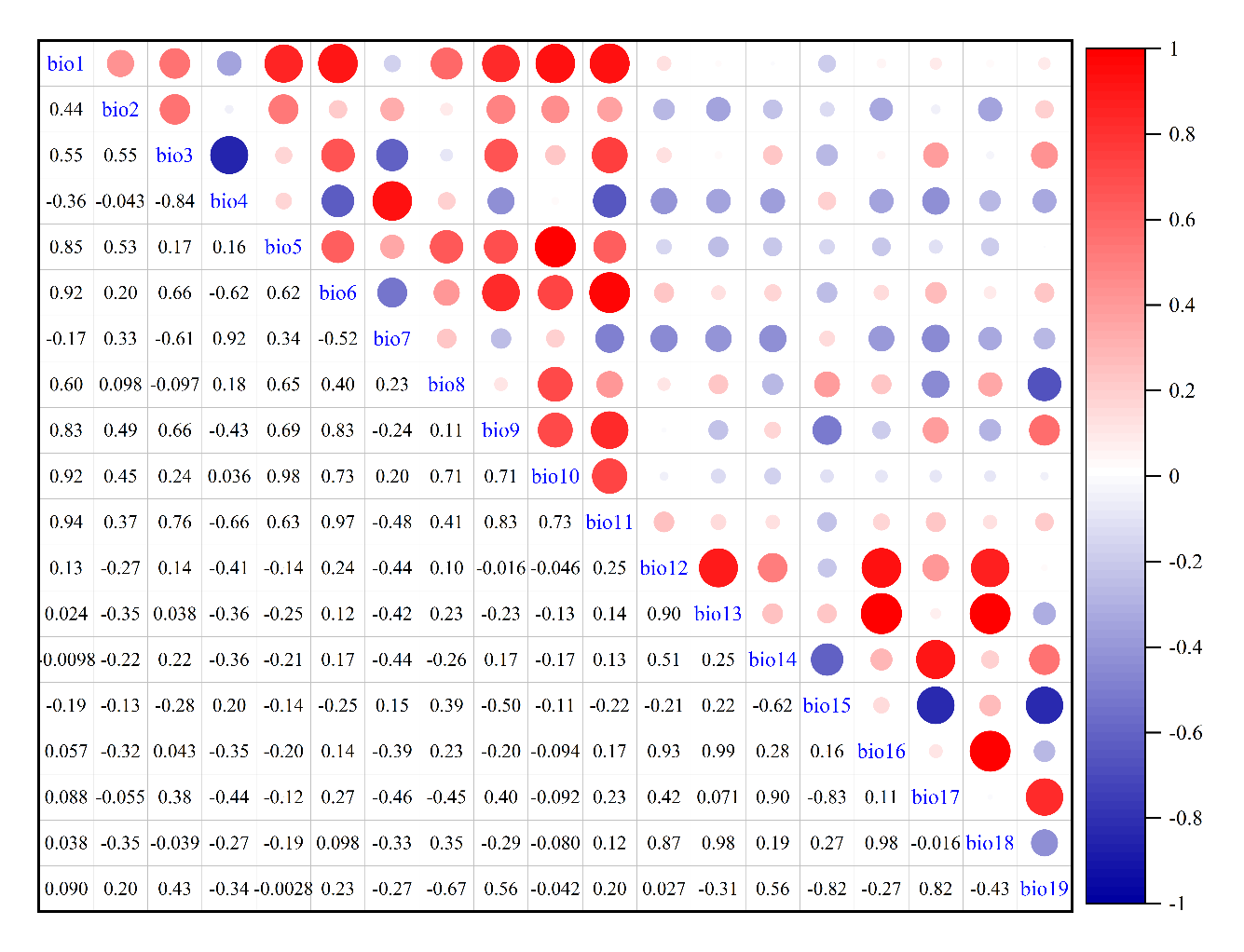


Fig.S26 Correlation among 19 climate variables (*Aster poliothamnus*)


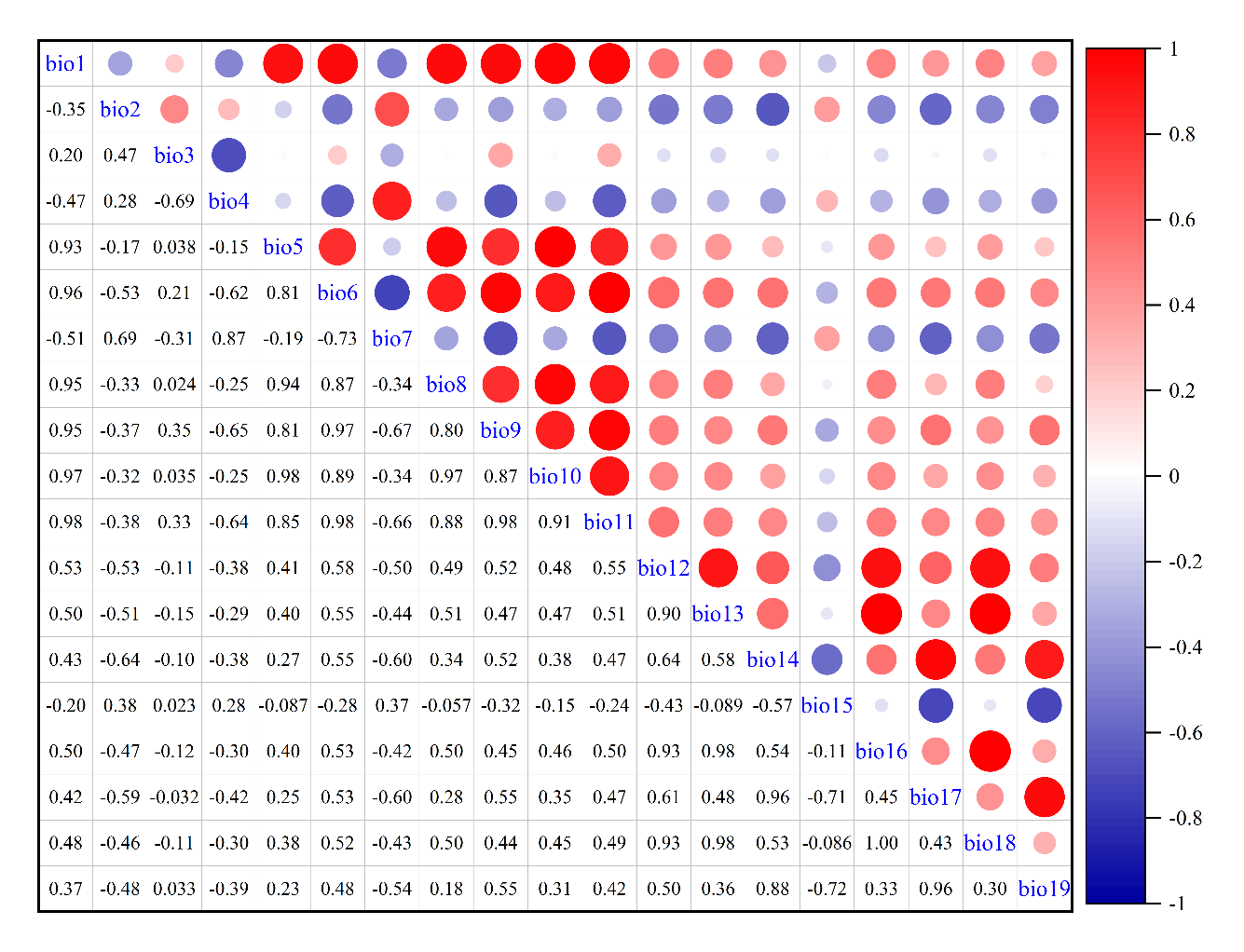


Fig.S27 Correlation among 19 climate variables (*Aster souliei*)


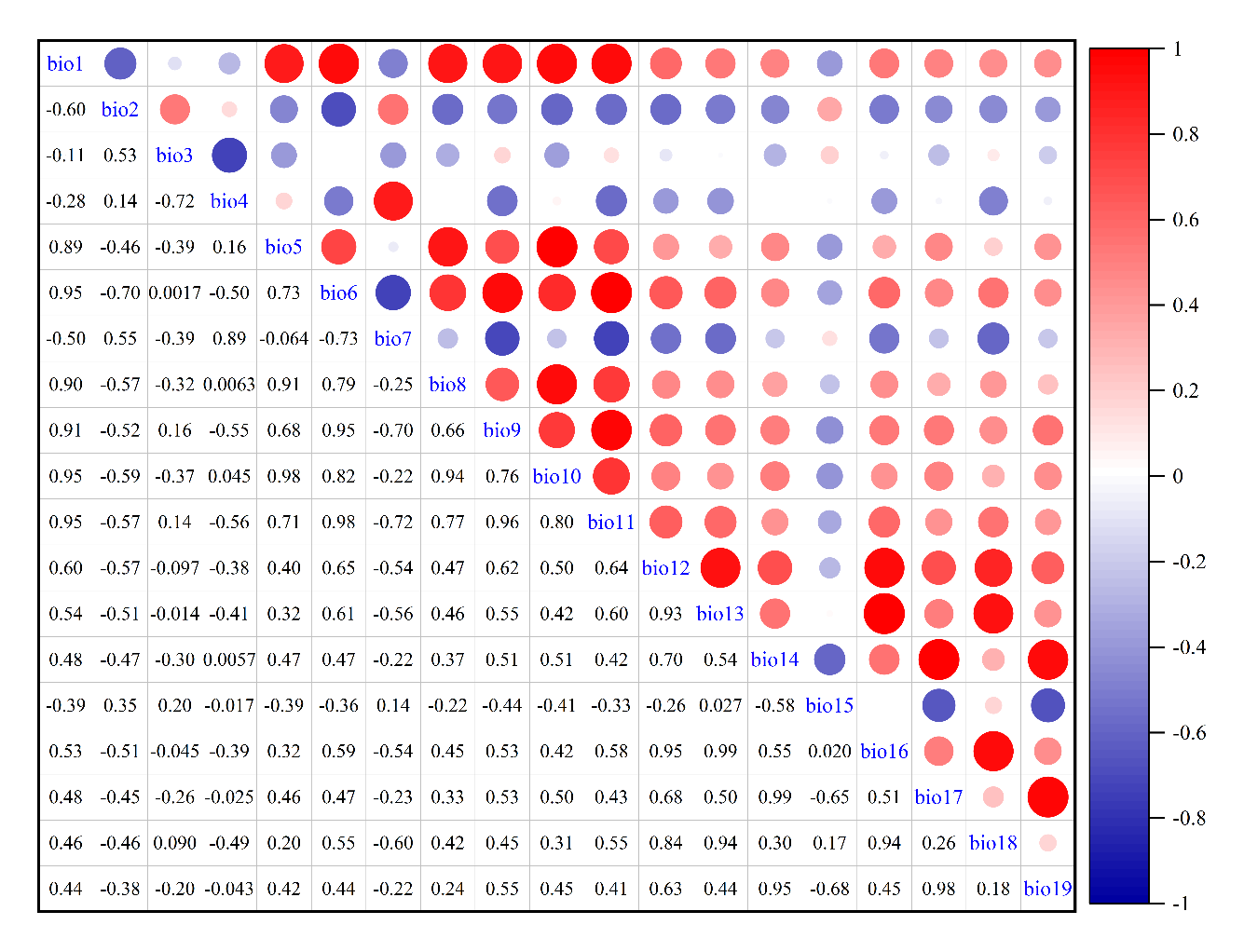


Fig.S28 Correlation among 19 climate variables (*Aster tongolensis*)


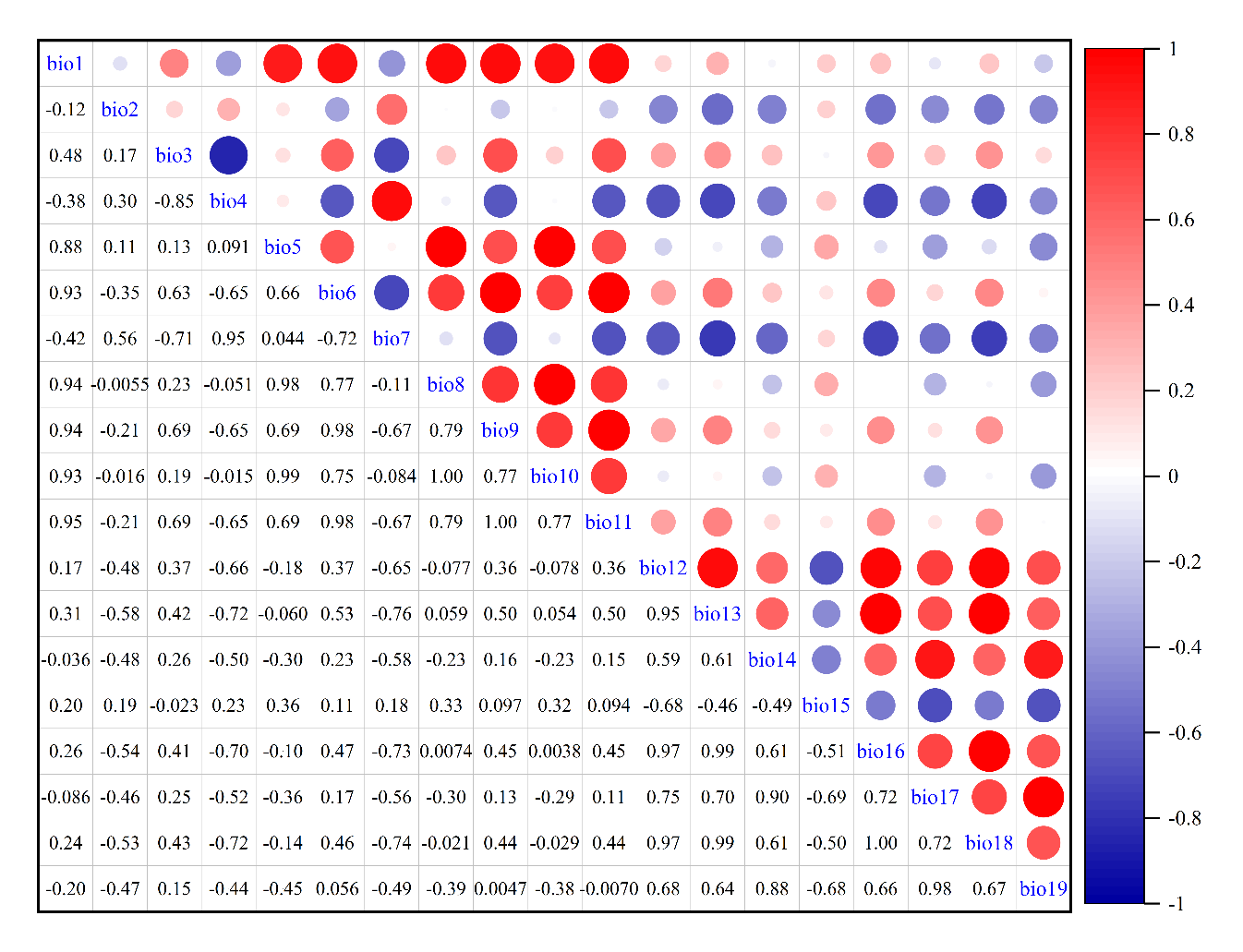


Fig.S29 Correlation among 19 climate variables (*Aster yunnanensis* var*. labrangensis*)
