# Supplementary material for: Prediction of Potential Distribution of Seven Plant Species of Aster (Asteraceae) Based on MaxEnt Model
Source: Ecol Evol. 2025 Sep 30;15(10):e71931. doi: 10.1002/ece3.71931 (PMC12483838; doi:10.1002/ece3.71931)
Supplement: Supplementary file 2 — Data S2: ece371931‐sup‐0002‐DataS2.docx. [file ECE3-15-e71931-s001.docx]

| Table S1 Number of occurrence records for each *Aster* species across data sources | | | | | |
| --- | --- | --- | --- | --- | --- |
| Species | Field Investigation (2020-2023) | China Digital Specimen Museum | GBIF | Literature and Flora | Total |
| *Aster asteroides* | 3 | 6 | 51 | 1 | 61 |
| *Aster diplostephioides* | 18 | 50 | 48 | 1 | 117 |
| *Aster farreri* | 21 | 22 | 86 | 2 | 131 |
| *Aster poliothamnus* | 3 | 15 | 56 | 1 | 75 |
| *Aster souliei* | 9 | 66 | 134 | 1 | 210 |
| *Aster tongolensis* | 5 | 34 | 87 | 2 | 128 |
| *Aster yunnanensis* var*. labrangensis* | 9 | 26 | 48 | 1 | 84 |
| Total | 68 | 219 | 510 | 9 | 806 |

| Table S2 Climate variables | |
| --- | --- |
| Variables | Description |
| Bio 1 | Annual Mean Temperature. |
| Bio 2 | Mean Diurnal Range. |
| Bio 3 | Isothermality (Bio 2/ Bio 7). (×100). |
| Bio 4 | Temperature Seasonality (standard. deviation ×100). |
| Bio 5 | Max Temperature of Warmest Month. |
| Bio 6 | Min Temperature of Coldest Month. |
| Bio 7 | Temperature Annual Range (Bio 5- Bio 6). |
| Bio 8 | Mean Temperature of Wettest Quarter. |
| Bio 9 | Mean Temperature of Driest Quarter. |
| Bio 10 | Mean Temperature of Warmest Quarter |
| Bio 11 | Mean Temperature of Coldest Quarter. |
| Bio 12 | Annual Precipitation. |
| Bio 13 | Precipitation of Wettest Month. |
| Bio 14 | Precipitation of Driest Month. |
| Bio 15 | Precipitation Seasonality. (Coefficient of Variation) |
| Bio 16 | Precipitation of Wettest Quarter. |
| Bio 17 | Precipitation of Driest Quarter. |
| Bio 18 | Precipitation of Warmest Quarter. |
| Bio 19 | Precipitation of Coldest. Quarter. |

| Table S3 Environment data description list | | | |
| --- | --- | --- | --- |
| Species | Variable | Percent contribution% | Permutation importance |
| *Aster asteroides* | Bio 10 | 66.3 | 38.6 |
|  | Bio 3 | 17.7 | 22 |
|  | Bio 12 | 13.3 | 34.2 |
|  | Bio 8 | 1.1 | 0.8 |
|  | Bio 19 | 0.6 | 2.5 |
|  | Bio 14 | 0.6 | 1.1 |
|  | Bio 2 | 0.3 | 0 |
|  | Bio 6 | 0.1 | 0.8 |
| *Aster diplostephioides* | Bio 3 | 55.9 | 19 |
|  | Bio 18 | 25.9 | 39.1 |
|  | Bio 10 | 16.4 | 36.5 |
|  | Bio 15 | 1.4 | 4.2 |
|  | Bio 2 | 0.2 | 0.9 |
|  | Bio 14 | 0.2 | 0.3 |
| *Aster farreri* | Bio 10 | 33.6 | 5.6 |
|  | Bio 9 | 21 | 12.9 |
|  | Bio 2 | 13.2 | 13.5 |
|  | Bio 12 | 11.6 | 34.8 |
|  | Bio 14 | 8.4 | 26 |
|  | Bio 15 | 7.1 | 4.9 |
|  | Bio 3 | 5.1 | 2.2 |
| *Aster poliothamnus* | Bio 4 | 31.9 | 29.9 |
|  | Bio 10 | 26.8 | 6.6 |
|  | Bio 11 | 14.8 | 19.8 |
|  | Bio 12 | 13.6 | 15.2 |
|  | Bio 2 | 6.4 | 20.4 |
|  | Bio 15 | 4.7 | 5.1 |
|  | Bio 3 | 1.4 | 2 |
|  | Bio 14 | 0.5 | 0.9 |
| *Aster souliei* | Bio 3 | 41.5 | 0.4 |
|  | Bio 4 | 23 | 73.6 |
|  | Bio 12 | 17.5 | 9 |
|  | Bio 10 | 13.9 | 2.4 |
|  | Bio 2 | 2.9 | 11.8 |
|  | Bio 15 | 1 | 0.4 |
|  | Bio 14 | 0.3 | 2.3 |
| *Aster tongolensis* | Bio 4 | 27.9 | 12.7 |
|  | Bio 3 | 20.2 | 2.3 |
|  | Bio 12 | 19 | 27.6 |
|  | Bio 11 | 15.5 | 35.7 |
|  | Bio 10 | 9.9 | 1.5 |
|  | Bio 2 | 5.2 | 12.7 |
|  | Bio 19 | 1.4 | 5.9 |
|  | Bio 15 | 0.8 | 1.5 |
| *Aster yunnanensis* var. *labrangensis* | Bio 3 | 27.1 | 2.9 |
|  | Bio 12 | 26.2 | 52.5 |
|  | Bio 10 | 21.4 | 7.5 |
|  | Bio 2 | 15.7 | 12.4 |
|  | Bio 15 | 7.4 | 4.1 |
|  | Bio 14 | 1.5 | 19.5 |
|  | Bio 6 | 0.7 | 1 |

| Table S4 Threshold values for binary habitat classification in species distribution models | | | |
| --- | --- | --- | --- |
| **Species** | Minimum training presence Logistic threshold | Maximum training sensitivity plus specificity Logistic threshold | Maximum test sensitivity plus specificity Logistic threshold |
| *Aster asteroides* | 0.0837 | 0.3969 | 0.3395 |
| *Aster diplostephioides* | 0.0642 | 0.2585 | 0.2718 |
| *Aster farreri* | 0.0094 | 0.2117 | 0.2413 |
| *Aster poliothamnus* | 0.0153 | 0.2605 | 0.2533 |
| *Aster souliei* | 0.0124 | 0.1547 | 0.144 |
| *Aster tongolensis* | 0.0104 | 0.223 | 0.2141 |
| *Aster yunnanensis* var. *labrangensis* | 0.121 | 0.225 | 0.2508 |
